# Optical control of AMPA receptors using a photoswitchable quinoxaline-2,3-dione antagonist†
†Electronic supplementary information (ESI) available: Experimental procedures and characterisation data. See DOI: 10.1039/c6sc01621a



**DOI:** 10.1039/c6sc01621a

**Published:** 2016-08-24

**Authors:** David M. Barber, Shu-An Liu, Kevin Gottschling, Martin Sumser, Michael Hollmann, Dirk Trauner

**Affiliations:** a Department of Chemistry and Center for Integrated Protein Science , Ludwig Maximilians University Munich , Butenandtstraße 5-13 , 81377 Munich , Germany . Email: dirk.trauner@lmu.de; b Department of Biochemistry I – Receptor Biochemistry , Ruhr-Universität-Bochum , Bochum 44780 , Germany

## Abstract

AMPA receptors respond to the neurotransmitter glutamate and play a critical role in excitatory neurotransmission. They have been implicated in several psychiatric disorders and have rich pharmacology. Antagonists of AMPA receptors have been explored as drugs and one has even reached the clinic. We now introduce a freely diffusible photoswitchable antagonist that is selective for AMPA receptors and endows them with light-sensitivity. Our photoswitch, **ShuBQX-3**, is active in its dark-adapted *trans*-isoform but is significantly less active as its *cis*-isoform. **ShuBQX-3** exhibits a remarkable red-shifting of its photoswitching properties through interactions with the AMPA receptor ligand binding site. Since it can be used to control action potential firing with light, it could emerge as a powerful tool for studying synaptic transmission with high spatial and temporal precision.

## Abstract 2

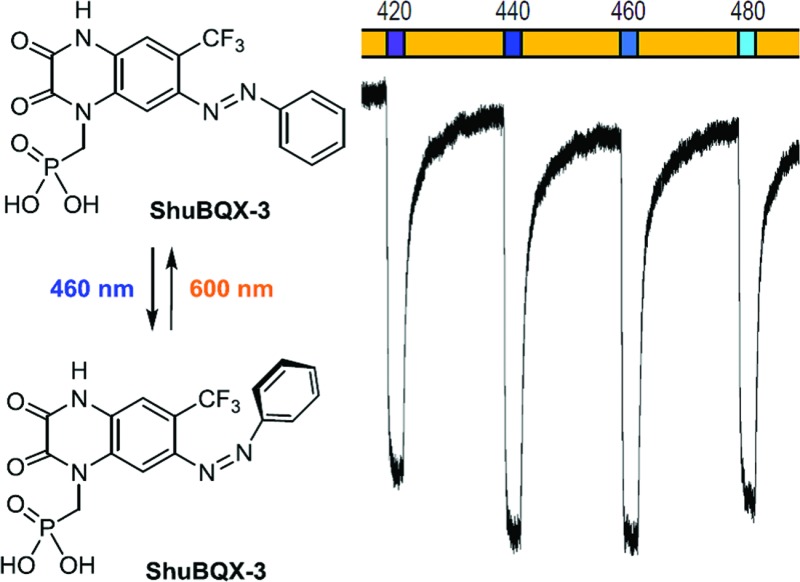
We have developed the first photoswitchable AMPA receptor antagonist, termed **ShuBQX-3**. It permits the precise optical control of AMPA receptors and exhibits a remarkable red-shifting of its photoswitching properties when bound to the receptor.

## Introduction

Photopharmacology is the attempt to endow biological targets with light sensitivity using small photoswitchable molecules.^1^ It has been applied to a wide variety of molecular targets, including ion channels,^2^ G-protein coupled receptors (GPCRs)^3^ and enzymes.^4^ As such, it has enabled the light-dependent control of diverse cellular processes, such as proliferation^5^ and neuronal excitability.^6^

The ionotropic glutamate receptors (iGluRs) are attractive targets for photopharmacology due to their fundamental roles in excitatory neurotransmission^7^ and their involvement in neurodegenerative conditions and psychiatric disorders.^8^ The iGluRs are natively gated by the neurotransmitter glutamate and are divided into three subclasses due to their individual responses to selective agonists: AMPA receptors (GluAs), kainate receptors (GluKs) and NMDA receptors (GluNs).^9^

To date, all three of the iGluR subtypes have been addressed with photopharmacology, using freely diffusible photoswitchable agonists.^10^ Although synthetic agonists for neurotransmitter receptors are powerful tools, they do create a non-physiological situation that can complicate the analyses of neural networks. This is perhaps the reason why antagonists of glutamatergic signaling are more widely used in neuroscience and why they have undergone extensive development as drugs to treat psychiatric diseases.^11^ It would be advantageous to use light to precisely control glutamate receptor antagonists and target their actions to specific locations. To that end, a few caged antagonists of iGluRs have been disclosed^12^ but their activation is irreversible. This prompted us to develop a photoswitchable antagonist that can be reversibly turned on and off. Our studies resulted in a quinoxaline-2,3-dione derivative, termed **ShuBQX-3**,^§^
§**ShuBQX** is a combination of the names of the authors D. M. B. and S. L. and the antagonist **MPQX**. that enables the optical control of AMPA receptor-mediated action potential firing of hippocampal neurons.

## Results and discussion

**Fig. 1:**
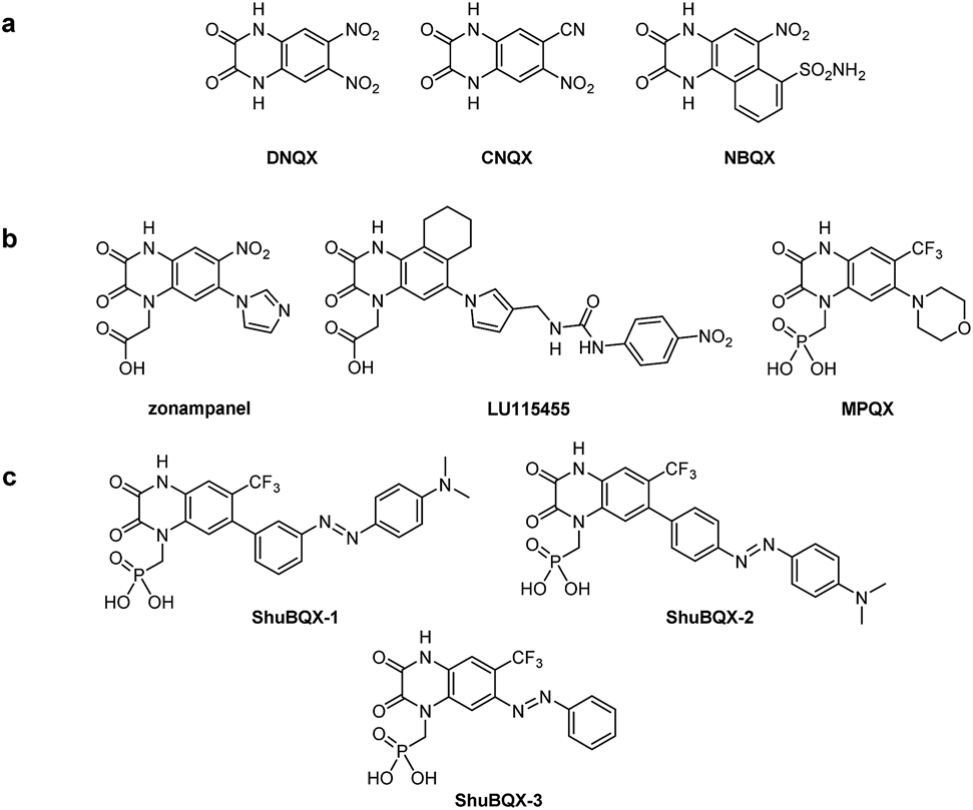
(a) Molecular structures of quinoxaline-2,3-dione AMPA receptor antagonists. (b) Molecular structures of quinoxaline-2,3-dione AMPA receptor antagonists with increased solubility. (c) Molecular structures of the designed photochromic AMPA receptor antagonists **ShuBQX-1**, **ShuBQX-2** and **ShuBQX-3**.

Our design of **ShuBQX-3** was based on the vast array of antagonists for AMPA receptors that have been developed.^13^ These encompass compounds that exhibit non-competitive antagonism, such as perampanel,^14^ which is clinically used, as well as those that compete for the glutamate binding site. Competitive AMPA receptor antagonists that contain the quinoxaline-2,3-dione motif (Fig. 1a and b), are an extremely well developed family of antagonists and have undergone extensive structure–activity relationship (SAR) studies.^15^

Starting from the parent compound **DNQX**, a wide array of more selective and more soluble derivatives have been developed, including **CNQX** and **NBQX** (Fig. 1a). To increase solubility, carboxylate or phosphonate moieties were introduced on the nitrogen in position 4, which gave rise to **zonampanel**, **LU115455** and **MPQX** (fanampanel) (Fig. 1b).^14^

We postulated that an azobenzene moiety could be accommodated in the 6-position of the quinoxaline-2,3-dione core as sterically bulky substituents are tolerated in this position. Our design hypothesis was also supported by a X-ray crystal structure of the compound **MPQX** bound to a GluA2 receptor.^16^ The crystal structure strongly suggested that a reasonable amount of steric bulk could be accommodated in the 6-position of the quinoxaline-2,3-dione without interfering with the binding of the antagonist. On the other hand, we concluded that photoisomerization of such a moiety would change the affinity of a ligand bound to the clamshell-like ligand binding domain. At the very least, this could be mediated by a reorganization of the solvation sphere upon photoisomerization. We also added the phosphonic acid side chain from the antagonist **MPQX** as it dramatically improves the solubility of the compound in aqueous solutions.^17^ When considering all of our strategic aspects, the photoswitchable antagonists **ShuBQX-1**, **ShuBQX-2** and **ShuBQX-3** were designed (Fig. 1c).

**Scheme 1:**
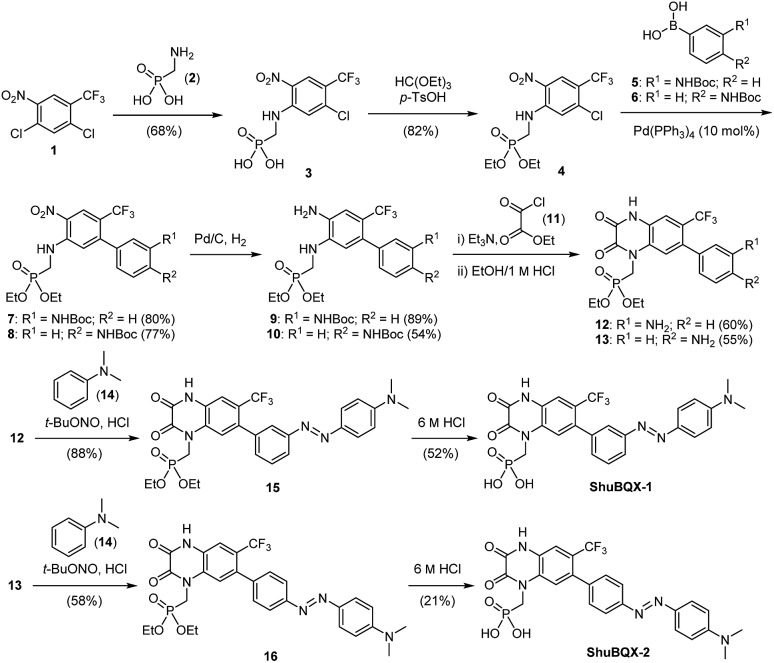
Synthesis of the photochromic AMPA receptor antagonists **ShuBQX-1** and **ShuBQX-2**.

We then set about preparing our target photoswitches, initially focusing on **ShuBQX-1** and **ShuBQX-2** (Scheme 1). Starting from dichloride **1** and aminomethylphosphonic acid (**2**) a S_N_Ar reaction furnished phosphonic acid **3**, which was protected to afford phosphonate ester **4**. Suzuki coupling reactions using boronic acids **5** and **6**, bearing Boc-protected anilines in the *meta*- and *para*-positions, respectively, were performed to provide biaryls **7** and **8**. Subsequent reduction of the nitro group followed by cyclization with ethyl chlorooxoacetate (**11**) afforded the desired *meta*- and *para*-amino substituted quinoxaline-2,3-diones **12** and **13**. Quinoxaline **12** was then converted to *meta*-azobenzene **15** in an azo-coupling reaction with *N*,*N*-dimethylaniline (**14**) and **ShuBQX-1** was furnished after deprotection of the phosphonate ester using 6 M HCl followed by reverse phase chromatography. The preparation of **ShuBQX-2** followed the same azo-coupling and deprotection procedures as **ShuBQX-1**, with moderate yields of the desired products being obtained in both reactions.

**Scheme 2:**
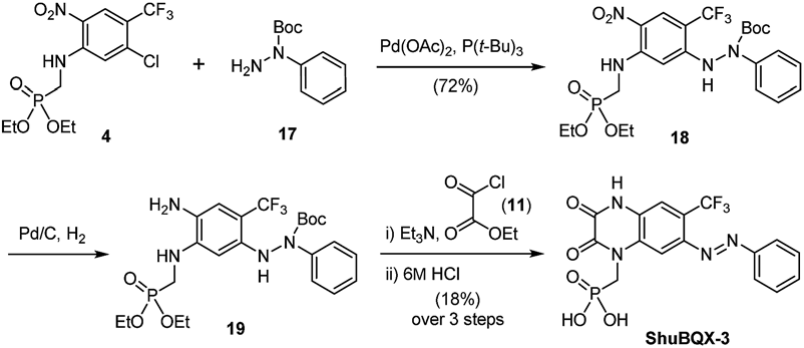
Synthesis of the photochromic AMPA receptor antagonist **ShuBQX-3**.

We next accomplished the synthesis of **ShuBQX-3** starting from phosphonate ester **4** (Scheme 2). A Buchwald–Hartwig cross-coupling of *N*-Boc protected hydrazine **17** with phosphonate ester **4** afforded protected hydrazine **18** in 72% yield. Reduction of the nitro group followed by cyclization to the quinoxaline-2,3-dione using ethyl chlorooxoacetate (**11**) and subsequent deprotection of the phosphonate ester gave **ShuBQX-3** in 18% yield (over three steps).

**Fig. 2:**
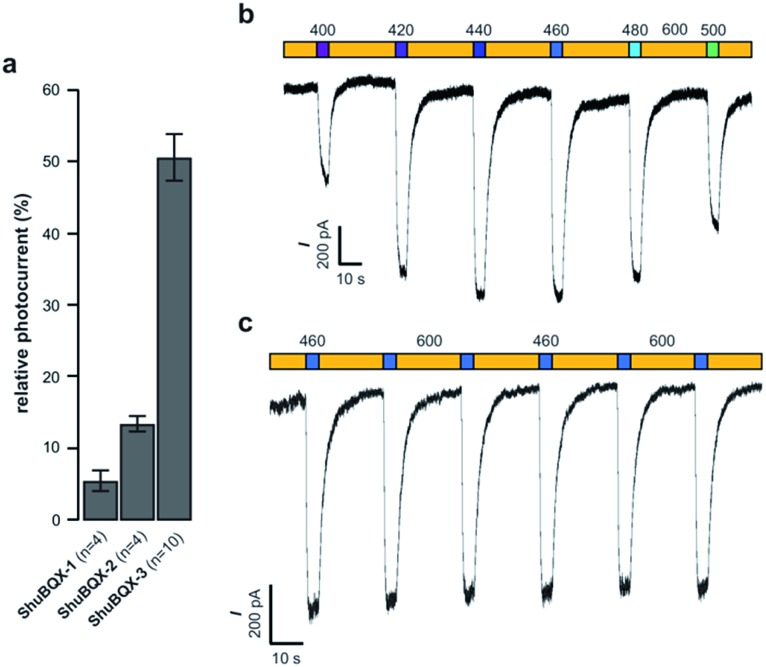
Whole-cell patch-clamp characterization of **ShuBQX-1**, **ShuBQX-2** and **ShuBQX-3** in the presence of glutamate (300 μM) using HEK293T cells transfected with GluA1-L497Y receptors. (a) Comparison of relative photocurrent of **ShuBQX-1**, **ShuBQX-2** and **ShuBQX-3** (5 μM). Values represent mean ± SEM. (b) Action spectrum of **ShuBQX-3** (20 μM) under illumination with orange light (600 nm) and varying wavelengths (400–500 nm). (c) Photoswitching of **ShuBQX-3** (10 μM) using 460 nm and 600 nm light over multiple switching cycles. Traces representative of *n* = 5 cells.

With our small family of soluble, photochromic ligands in hand, we first deduced the optimum photoswitching wavelengths using UV-Vis spectroscopy (Fig. S1†) and then determined their functional properties as light-controllable AMPA receptor antagonists. Whole-cell patch-clamp electrophysiology of HEK293T cells expressing GluA1-L497Y receptors (a non-desensitizing AMPA receptor mutant)^18^ found that the photochromic ligands **ShuBQX-1** (5 μM) and **ShuBQX-2** (5 μM) are good antagonists of AMPA receptors in the presence of glutamate (300 μM). However, upon photoisomerization using blue (460 nm) and green (560 nm) light only a small change in AMPA receptor antagonism was observed (Fig. 2a). We then evaluated **ShuBQX-3** (5 μM) and discovered that it is an excellent photoswitchable antagonist of AMPA receptors.

First, we determined the IC_50_ value of **ShuBQX-3** in the absence of light and in the presence of glutamate (300 μM). It was found to be 3.1 μM (Fig. S3†). A very similar value (IC_50_ = 3.3 μM) was obtained when illuminating **ShuBQX-3** with orange light (600 nm). At this wavelength, the photoswitch largely resides in the *trans*-form. Under blue light illumination (460 nm), which favors the *cis*-state of **ShuBQX-3**, the antagonist was significantly less potent than in the dark-adapted state (Fig. 2). Finally, experiments conducted using **ShuBQX-3** with differing concentrations of glutamate (100 μM and 1 mM) confirmed that it is a competitive antagonist of GluA1 receptors (Fig. S4†). Further studies on the biological activity of **ShuBQX-3** using patch-clamp electrophysiology showed that glutamate-induced currents are completely blocked in its *trans*-form. Upon illumination with 460 nm light, 50% of the glutamate-induced current (compared to the current induced in the absence of **ShuBQX-3**) could be released (Fig. 2a). We then determined the action spectrum of **ShuBQX-3** in the presence of glutamate. When the wavelength was switched between orange light (600 nm) and different wavelengths of purple blue and green light (400–500 nm), we observed large differences in current (Fig. 2b). The maximum inward current was consistently observed when illuminating with 440 nm or 460 nm (Fig. S5†) whereas minimum inward currents were observed using long wavelength light or in the dark. Reversible photoactivation of AMPA receptors with **ShuBQX-3** is robust and could be repeated over many times without any significant loss of receptor antagonism (Fig. 2c). Highly reproducible photoswitching of **ShuBQX-3** was also obtained when operating in current-clamp mode (Fig. S6†).

**Fig. 3:**
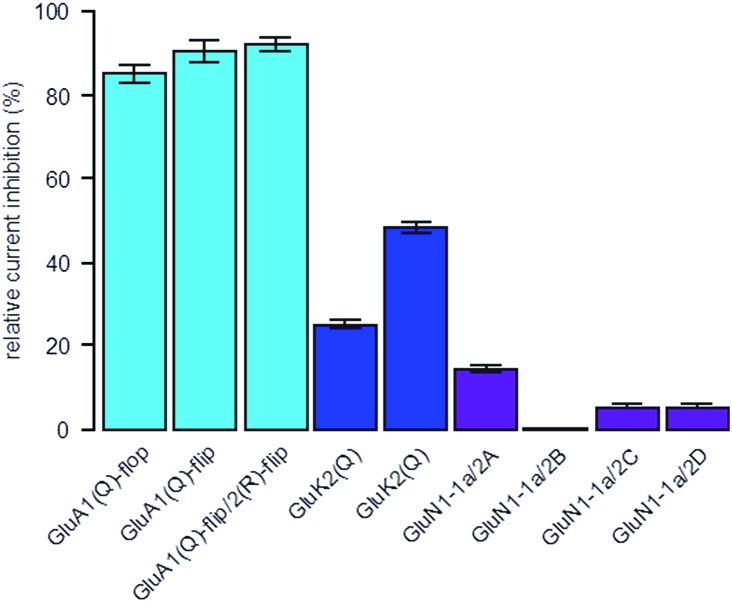
Selectivity profile of **ShuBQX-3** (5 μM) in the dark at various glutamate receptors expressed in *Xenopus* oocytes. Currents were induced using the following methods: GluA1-containing receptors = kainic acid (100 μM); GluK2 – containing receptors = glutamate (100 μM, left column) and glutamate (30 μM, right column); GluN1-1a-containing receptors = glutamate (100 μM) and glycine (10 μM). Values represent mean ± SEM (*n* = 5 oocytes).

To determine the selectivity profile of **ShuBQX-3** (5 μM) in its dark state, we evaluated its effects on *Xenopus* oocytes expressing a variety of glutamate receptors (Fig. 3). **ShuBQX-3** (5 μM) showed excellent antagonism of all GluA1-containing receptors that were tested (85–93%). By contrast, the amount of antagonism observed at this concentration on the GluK2 receptor was significantly reduced (25%) when inducing the current using glutamate (100 μM). Reducing the concentration of glutamate (30 μM) increased the amount of antagonism exhibited by **ShuBQX-3** at the GluK2 receptor (49%). Thus, indicating that the dark state of **ShuBQX-3** is a competitive antagonist of the GluK2 receptor. Additionally, **ShuBQX-3** (5 μM) was evaluated against several GluN1-1a-containing receptors. **ShuBQX-3** (5 μM) displayed minimal antagonism at the GluN1-1a-containing glutamate receptors, demonstrating that **ShuBQX-3** is partially selective for AMPA receptors over kainate, whilst having significantly reduced levels of antagonism at NMDA receptors.

**Fig. 4:**
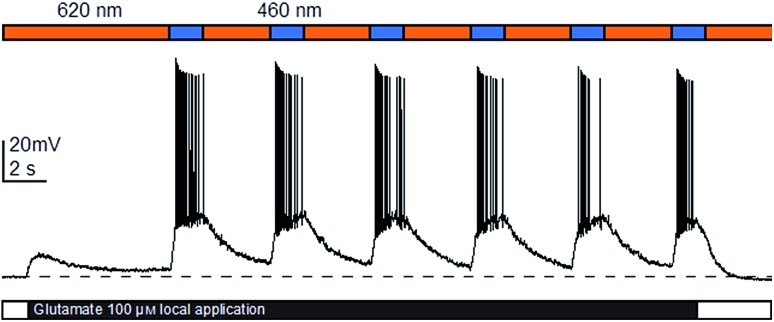
Optical control of action potential firing in hippocampal CA1 neurons using **ShuBQX-3** (10 μM) in the presence of glutamate (100 μM) and AP-5 (50 μM).

With the full evaluation of **ShuBQX-3** in transfected HEK293T cells and *Xenopus* oocytes complete, we set out to demonstrate that **ShuBQX-3** could control native AMPA receptors in excitable cells. For these experiments we used acute mouse brain slice preparations and whole cell patch-clamp electrophysiology of hippocampal CA1 neurons. Pleasingly, when **ShuBQX-3** (10 μM) and glutamate (100 μM) were locally applied in a brain slice preparation, the induced action potential firing of a single neuron could be effectively controlled by switching between blue (460 nm) and orange (620 nm) light (Fig. 4). The NMDA-receptor antagonist AP-5 (50 μM) was locally added to ensure that the action potential firing was not caused by any interactions between the NMDA receptors and glutamate. Additionally, we were able to optically control hippocampal CA1 neurons when using AMPA as the agonist (Fig. S7†). Demonstrating that **ShuBQX-3** is selective for AMPA receptors.

**Fig. 5:**
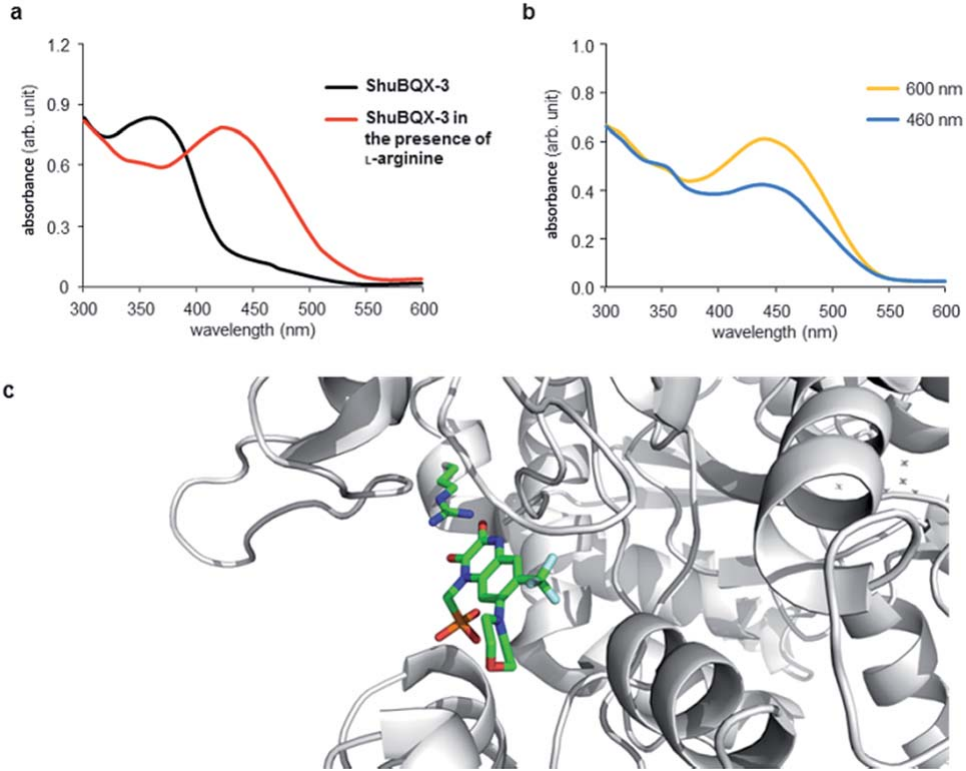
UV-Vis analysis of the photoswitching properties of **ShuBQX-3**. (a) UV-Vis spectrum showing **ShuBQX-3** (50 μM in DMSO) and the bathochromic shift in the presence of 1 mM l-arginine. (b) UV-Vis spectrum **ShuBQX-3** (50 μM) in DMSO in the presence of 1 mM l-arginine when illuminated with 460 nm and 600 nm light. (c) X-ray structure of **MPQX** bound to a GluA2 receptor ligand binding domain, showing the interaction between a conserved arginine and the quinoxaline-2,3-dione.^16^

The action spectrum of **ShuBQX-3** is noteworthy, as we observed a significant difference between the optimal photoswitching wavelengths established by the UV-Vis experiments and the physiological patch-clamp experiments. In our UV-Vis experiments, a solution of **ShuBQX-3** (50 μM in DMSO) displayed optimal photoswitching at 380 nm (*trans* to *cis*) and 460 nm (*cis* to *trans*), with a *λ*_max_ = 365 nm for the *trans*-isomer (Fig. 5a, S1†). However, in the patch-clamp experiments in HEK293T cells expressing the GluA1 receptor, **ShuBQX-3** exhibited a bathochromic shift in its action spectrum, with optimal photoswitching now taking place when illuminating with 460 nm and 600 nm light.^19^ In an attempt to ascertain why this bathochromic shift was occurring, we consulted the X-ray crystal structure of the non-photoswitchable antagonist **MPQX** bound to a GluA2 receptor (Fig. 5c).^16^ The structure features prominent interaction between the guanidinium moiety of arginine R-485, which is also involved in glutamate binding, and the quinoxaline-2,3-dione core of **MPQX**. We therefore postulated that this interaction could have an effect on the photoswitching properties of **ShuBQX-3** when it is bound to the GluA1 receptor.

To probe this possible interaction, we took the UV-Vis solution of **ShuBQX-3** (50 μM in DMSO) and doped it with l-arginine. Indeed, a bathochromic shift of 75 nm (*λ*_max_ = 440 nm) in the UV-Vis spectrum of **ShuBQX-3** was observed (Fig. 5a) with a 20-fold excess of l-arginine. The optimum photoswitching wavelengths of **ShuBQX-3** were now demonstrated to be 460 nm and 600 nm (Fig. 5b). When l-arginine was replaced by guanidine, a similar bathochromic shift was observed (Fig. S8†), substantiating our hypothesis. UV-Vis experiments conducted with the diethyl phosphonate of **ShuBQX-3** showed that the phosphonic acid side chain is not crucial for the interaction (Fig. S9†). We also measured the thermal relaxation rate of **ShuBQX-3** in the presence of l-arginine (*τ* = 1.68 min) and found it to be faster than the rate of **ShuBQX-3** alone (*τ* = 7.37 min). This is in accordance to the rate acceleration of red-shifted azobenzenes in comparison to their non red-shifted analogues.^20^ To the best of our knowledge, this is the first example of an azobenzene-based photoswitch to exhibit such red-shifting properties. Control experiments using **ShuBQX-3** (50 μM) dissolved in Ringer's solution showed almost no change in the absorption maximum (Fig. S1†). Our findings suggest that the interaction between R-485 and the quinoxaline-2,3-dione is responsible for the bathochromic shift in the action spectrum of **ShuBQX-3**.

## Conclusions

In summary, we have developed a photochromic antagonist that permits the precise optical control of AMPA receptors. Our photoswitch, **ShuBQX-3**, is active as its *trans*-isomer and is converted to its *cis*-isomer using blue light illumination enabled by red-shifting upon binding to AMPA receptors. **ShuBQX-3** expands the photopharmacology of iGluRs and demonstrates that potent photoswitchable antagonists for these receptors can be developed. We envision that **ShuBQX-3** will be an important tool for studying the function of AMPA receptors *in vivo*.

## Live subject statement

All animal procedures were performed in accordance with the guidelines of the Regierung Oberbayern/the Tierschutzgesetz (TierSchG) and the TierschutzVersuchstierverordnung (TierSchVersV).
