# Supplementary material for: Optical control of AMPA receptors using a photoswitchable quinoxaline-2,3-dione antagonist
Source: Chem Sci. 2016 Aug 24;8(1):611–5. doi: 10.1039/c6sc01621a (PMC5358534; doi:10.1039/c6sc01621a)
Supplement: Supplementary file 1 [file SC-008-C6SC01621A-s001.pdf]

Electronic Supplementary Material (ESI) for Chemical Science.
This journal is © The Royal Society of Chemistry 2016

Electronic Supplementary Information

Optical Control of AMPA Receptors Using a Photoswitchable Quinoxaline-2,3-dione Antagonist

David M. Barber,[a] Shu-An Liu,[a] Kevin Gottschling,[b] Martin Sumser,[a] Michael Hollmann[b] and Dirk Trauner*[a]

[a] *Department of Chemistry and Center for Integrated Protein Science, Ludwig Maximilians University Munich, Butenandtstraße 5-13, 81377 Munich, Germany*

[b] *Department of Biochemistry I – Receptor Biochemistry, Ruhr-Universität-Bochum, Bochum 44780, Germany*

dirk.trauner@lmu.de

Index

General Experimental Details for Chemical Synthesis	3
Synthetic Procedures	6
NMR Spectra	22
Supplementary Figures	38
HEK293T Cell Electrophysiology	42
Brain Slice Electrophysiology	42
Xenopus Oocytes Electrophysiology	43

General Experimental Details for Chemical Synthesis

General Experimental Techniques. All reactions were conducted using oven-dried glassware (120 °C) under a positive pressure of nitrogen with magnetic stirring unless otherwise stated. Liquid reagents and solvents were added via syringe or oven-dried stainless steel cannulas through rubber septa. Solids were added under inert gas counter flow or were dissolved in specified solvents prior to addition. Low temperature reactions were carried out in a Dewar vessel filled with the appropriate cooling agent e.g. H_2O/ice (0 °C). Reactions using temperatures above room temperature were conducted using a heated oil bath. Yields refer to spectroscopically pure compounds unless otherwise stated.

Solvents and Reagents. Tetrahydrofuran (THF) was distilled under a nitrogen atmosphere from Na/benzophenone prior to use. Triethylamine (Et_3N) was distilled under a nitrogen atmosphere from CaH_2 prior to use. Dry dichloromethane (CH_2Cl_2), toluene (PhMe) and methanol (MeOH) were purchased from commercial suppliers and used as received. Solvents for extraction and flash column chromatography were purchased in technical grade purity and distilled under reduced pressure prior to use. All other reagents and solvents were purchased from commercial suppliers and used as received.

Chromatography. Reactions and chromatography fractions were monitored by qualitative thin-layer chromatography (TLC) on silica gel F_{254} TLC plates from Merck KGaA. Analytes on the glass plates were visualized by irradiation with UV light and by immersion of the TLC plate in an appropriate staining solution followed by heating with a hot-air gun. The following staining solutions were applied: Hanessian's (CAM) staining solution [$Ce(SO_4)_2$ (5.0 g), $(NH_4)_6Mo_7O_{24}\cdot 4H_2O$ (25 g), concentrated aqueous H_2SO_4 (50 mL) and H_2O (450 mL)]; potassium permanganate staining solution [$KMnO_4$ (3.0 g), K_2CO_3 (20 g), 5% aqueous NaOH (5.0 mL) and H_2O (300 mL)]; ninhydrin staining solution [ninhydrin (20.0 g) and ethanol (600 mL)]. Flash column chromatography was performed using silica gel, particle size 40–63 µm (eluents are given in parenthesis).

Compound Naming. Compound names were generated using the PerkinElmer software ChemDraw according to standard IUPAC nomenclature.

Melting Points. Melting points were measured on an EZ-Melt apparatus from Stanford Research Systems and are uncorrected.

NMR Spectroscopy. NMR spectra were measured on a Bruker Avance III HD 400 MHz spectrometer equipped with a CryoProbeTM operating at 400 MHz for proton nuclei, 100 MHz for carbon nuclei, 376 MHz for fluorine nuclei and 162 MHz for phosphorus nuclei. The ^{1}H and ^{13}C NMR shifts are reported in ppm related to the chemical shift of tetramethylsilane. ^{1}H NMR shifts were calibrated to residual solvent resonances: $CDCl_3$ (7.26 ppm), CD_3OD (3.31 ppm) and $(CD_3)_2SO$ (2.50 ppm). ^{13}C NMR shifts were calibrated to the centre of the multiplet signal of the residual solvent resonance: $CDCl_3$ (77.16 ppm), CD_3OD (49.00 ppm) and $(CD_3)_2SO$ (39.52 ppm). The ^{19}F NMR shifts are reported in ppm related to the chemical shift of trichlorofluoromethane. The ^{31}P NMR shifts are reported in ppm related to the chemical shift of 85% phosphoric acid. ^{1}H NMR spectroscopic data are reported as follows: Chemical shift in ppm (multiplicity, coupling constants, integration). The multiplicities are abbreviated with s (singlet), br s (broad singlet), d (doublet), t (triplet), q (quartet) and m (multiplet). Except for multiplets, the chemical shift of all signals is reported as the centre of the resonance range. Additionally to ^{1}H and ^{13}C NMR measurements, 2D NMR techniques such as homonuclear correlation spectroscopy (COSY), heteronuclear single quantum coherence (HSQC) and heteronuclear multiple bond coherence (HMBC) were used to assist the compound identification process. Coupling constants *J* are reported in Hz. All raw fid files were processed and the spectra analysed using the program MestReNova 9.0 from Mestrelab Research S. L.

Infrared Spectroscopy. IR spectra were recorded on a PerkinElmer Spectrum BX II FT-IR instrument equipped with an ATR unit. The measured wave numbers are reported in cm^{-1}.

Mass Spectrometry. All high-resolution mass spectra (HRMS) were recorded by the LMU Mass Spectrometry Service. HRMS were recorded on a MAT 90 (ESI) spectrometer from Thermo Finnigan GmbH.

UV-Vis Spectroscopy. UV-Vis spectra were recorded on a Varian Cary 50 Scan UV-Vis spectrometer using Helma SUPRASIL precision cuvettes (10 mm light path).

LCMS. LCMS was performed on an Agilent 1260 Infinity HPLC System, MS-Agilent 1100 Series, Type: 1946D, Model: SL, equipped with a Agilent Zorbax Eclipse Plus C18 (100 × 4.6 mm, particle size 3.5 micron) reverse phase column. Retention times (**t_R**) are given in minutes (min).

Synthetic Procedures

Synthesis and characterisation of ({[5-chloro-2-nitro-4-(trifluoromethyl)phenyl]amino} methyl)phosphonic acid (3)

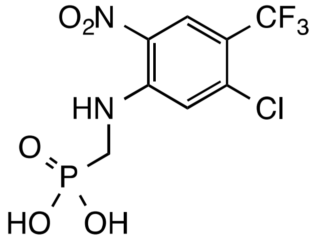

To a suspension of compound **2** (3.12 g, 12.0 mmol) in EtOH/H_2O (100 mL, 1:1) at r.t. was added compound **1** (1.29 g, 11.6 mmol) and Na_2CO_3 (1.32 g, 12.5 mmol). The resulting mixture was heated to 105 °C for 6 h. The reaction mixture was cooled to r.t. and the EtOH was removed under reduced pressure. The resulting mixture was diluted with H_2O (100 mL) and washed with EtOAc (100 mL). The aqueous phase was acidified with 1 M HCl (40 mL) and extracted with EtOAc (3 × 100 mL). The combined organic extracts were washed with H_2O (40 mL), dried over Na_2SO_4, filtered and concentrated under reduced pressure to afford compound **3** (1.37 g, 68%) as a yellow solid.

mp: 207 – 209 °C decomp; **^{1}H NMR** (400 MHz, CD_3OD): δ 8.47 (s, 1H), 7.39 (s, 1H), 3.82 (d, *J* = 13.0 Hz, 2H); **^{13}C NMR** (100 MHz, CD_3OD): δ 148.1 (d, *J* = 6.0 Hz), 139.9, 131.2, 127.7 (q, *J* = 6.0 Hz), 124.0 (q, *J* = 270.5 Hz), 118.5, 116.0 (q, *J* = 33.0 Hz), 41.2 (d, *J* = 152.5 Hz); **^{19}F NMR** (376 MHz, CD_3OD): δ −62.7; **^{31}P NMR** (162 MHz, CD_3OD): δ 18.6; **IR** (neat): 3350, 2767, 1621, 1575, 1525, 1511, 1426, 1413, 1353, 1301, 1272, 1234, 1209, 1176, 1131, 1114, 1073, 1014, 946, 917, 877, 846, 742, 702 cm^{-1}; **HRMS** (ESI, *m/z*): [$(M-H)^-$] calcd. for $C_8H_6ClF_3N_2O_5P^-$, 332.9660; found 332.9665.

Synthesis and characterisation of diethyl ({[5-chloro-2-nitro-4-(trifluoromethyl)phenyl] amino}methyl)phosphonate (4)

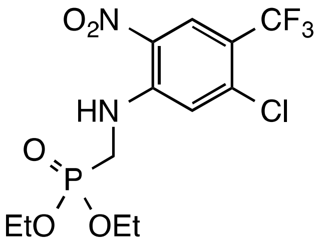

To a solution of compound **3** (1.67 g, 5.00 mmol) in triethyl orthoformate (22.3 g, 150 mmol, 25 mL) at r.t. was added *p*-TsOH (190 mg, 1.00 mmol). The resulting mixture was heated to 150 °C and stirred for 3.5 h. The reaction mixture was cooled to r.t. and concentrated under reduced pressure. The residue was diluted with H_2O (40 mL) and extracted with EtOAc (2 × 40 mL). The combined organic extracts were dried over Na_2SO_4, filtered and concentrated under reduced pressure. Purification by flash column chromatography on silica gel eluting with *i*-Hex/EtOAc (1:1) afforded compound **4** (1.61 g, 82%) as a yellow solid.

mp: 87 – 89 °C; **TLC** (*i*-Hex/EtOAc, 1:1): R_f = 0.20 (UV/$KMnO_4$); **^{1}H NMR** (400 MHz, $CDCl_3$): δ 8.51 (s, 1H), 8.50 – 8.41 (m, 1H), 7.09 (s, 1H), 4.27 – 4.12 (m, 4H), 3.71 (dd, *J* = 13.5, 6.0 Hz, 2H), 1.35 (t, *J* = 7.0 Hz, 6H); **^{13}C NMR** (100 MHz, $CDCl_3$): δ 146.4 (d, *J* = 6.5 Hz), 139.9, 130.2, 127.2 (q, *J* = 5.5 Hz), 122.3 (q, *J* = 271.5 Hz), 116.6, 116.5 (q, *J* = 33.5 Hz), 63.23 (d, *J* = 7.0 Hz), 39.6 (d, *J* = 157.8 Hz), 16.6 (d, *J* = 6.0 Hz); **^{19}F NMR** (376 MHz, $CDCl_3$): δ −61.6; **^{31}P NMR** (162 MHz, $CDCl_3$): δ 20.0; **IR** (thin film): 3361, 2985, 1621, 1579, 1530, 1435, 1354, 1317, 1302, 1243, 1205, 1157, 1126, 1049, 1024, 969, 945, 917, 897, 838, 781, 764, 702 cm^{-1}; **HRMS** (ESI, *m/z*): $[(M{-}H)^-]$ calcd. for $C_{12}H_{14}ClF_3N_2O_5P^-$, 389.0286; found 389.0296.

Synthesis and characterisation of diethyl [({3'-[(*tert*-butoxycarbonyl)amino]-4-nitro-6-(trifluoromethyl)biphenyl-3-yl}amino)methyl]phosphonate (7)

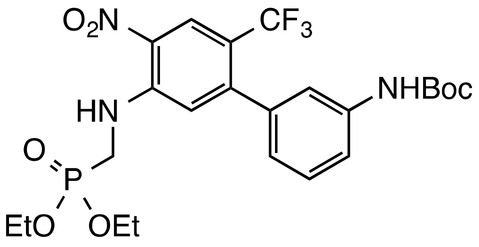

To a mixture of compound **4** (781 mg, 2.00 mmol) and 3-(*N*-boc-amino)phenylboronic acid (**5**) (522 mg, 2.20 mmol) in PhMe/EtOH (3.5:1, 45 mL) at r.t. under argon was added a solution of Na_2CO_3 (4 mL, 2.0 M in H_2O) and $Pd(PPh_3)_4$ (116 mg, 0.100 mmol). The resulting mixture was degassed and flushed with argon (× 3) and then heated to 95 °C for 17 h. The reaction mixture was cooled to r.t., concentrated under reduced pressure and the resulting residue was directly purified by flash column chromatography eluting with CH_2Cl_2/EtOAc (4:1) to afford compound **7** (873 mg, 80%) as a yellow oil.

TLC (CH_2Cl_2/EtOAc, 4:1): R_f = 0.41 (UV/ninhydrin); **^{1}H NMR** (400 MHz, $CDCl_3$): δ 8.57 (s, 1H), 8.45 (q, *J* = 6.0 Hz, 1H), 7.44 – 7.36 (m, 2H), 7.35 – 7.28 (m, 1H), 6.97 (d, *J* = 7.5 Hz, 1H), 6.83 (s, 1H), 6.64 (s, 1H), 4.18 (dq, *J* = 8.5, 7.0 Hz, 4H), 3.69 (dd, *J* = 13.5, 6.0 Hz, 2H), 1.51 (s, 9H), 1.33 (t, *J* = 7.0 Hz, 6H); **^{13}C NMR** (100 MHz, $CDCl_3$): δ 152.7, 148.6 (q, *J* = 1.5 Hz), 145.5 (d, *J* = 7.5 Hz), 139.0, 138.3, 130.8, 128.8, 126.1 (q, *J* = 6.0 Hz), 123.4 (q, *J* = 272.5 Hz), 123.0 (q, *J* = 1.5 Hz), 118.6, 118.4, 117.6, 117.4 (q, *J* = 32.0 Hz), 80.8, 63.1 (d, *J* = 7.0 Hz), 39.4 (d, *J* = 157.0 Hz), 28.4, 16.6 (d, *J* = 5.5 Hz); **^{19}F NMR** (376 MHz, $CDCl_3$): δ −56.6; **^{31}P NMR** (162 MHz, $CDCl_3$): δ 20.5; **IR** (thin film): 3355, 2981, 2931, 1720, 1625, 1609, 1575, 1532, 1486, 1427, 1346, 1305, 1268, 1235, 1153, 1122, 1048, 1019, 971, 794, 764, 735 cm^{-1}; **HRMS** (ESI, *m/z*): $[(M+Na)^+]$ calcd. for $C_{23}H_{29}F_3N_3NaO_7P^+$, 570.1587; found 570.1590.

Synthesis and characterisation of diethyl [({4-amino-3'-[(*tert*-butoxycarbonyl)amino]-6-(trifluoromethyl)biphenyl-3-yl}amino)methyl]phosphonate (9)

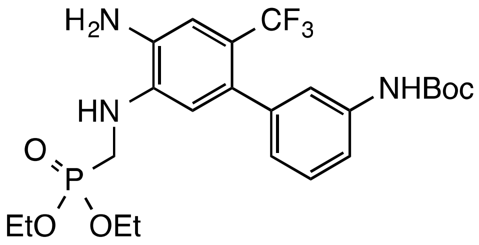

To a solution of compound **7** (1.04 g, 1.90 mmol) in MeOH (30 mL) at r.t. was added Pd/C (104 mg, 10 wt. % Pd labelling). The reaction vessel was flushed with hydrogen (× 3) and the resulting mixture was stirred at r.t. for 8 h. The reaction mixture was filtered through a pad of Celite® washing with MeOH (30 mL). The filtrate was concentrated under reduced pressure to afford compound **9** (876 mg, 89%) as a pale brown oil, which was used in the next step without further purification.

TLC (CH_2Cl_2/MeOH, 19:1): R_f = 0.27 (UV/ninhydrin); **^{1}H NMR** (400 MHz, CD_3OD): δ 7.42 – 7.31 (m, 2H), 7.25 – 7.18 (m, 1H), 7.05 (s, 1H), 6.93 (d, *J* = 7.5 Hz, 1H), 6.60 (s, 1H), 4.11 (dq, *J* = 8.0, 7.0 Hz, 4H), 3.69 (d, *J* = 10.5 Hz, 2H), 1.51 (s, 9H), 1.27 (dt, *J* = 7.0, 0.5 Hz, 6H); **^{13}C NMR** (100 MHz, CD_3OD): δ 155.3, 143.1, 139.9, 139.4 – 139.3 (m), 135.0, 133.9 (q, *J* = 2.0 Hz), 128.8, 126.4 (q, *J* = 271.5 Hz), 124.9 (q, *J* = 2.0 Hz), 121.1 – 120.8 (m), 118.7 (q, *J* = 30.0 Hz), 118.5 – 118.3 (m), 115.4 – 115.2 (m), 114.2 (q, *J* = 5.5 Hz), 80.8, 64.0 (d, *J* = 7.0 Hz), 40.3 (d, *J* = 158.5 Hz), 28.7, 16.7 (d, *J* = 5.5 Hz); **^{19}F NMR** (376 MHz, CD_3OD): δ −56.2; **^{31}P NMR** (162 MHz, CD_3OD): δ 25.0; **IR** (thin film): 3362, 2979, 2936, 1703, 1607, 1583, 1530, 1488, 1367, 1302, 1233, 1197, 1152, 1106, 1049, 1019, 973, 789 cm^{-1}; **HRMS** (ESI, *m/z*): [$(M-H)^-$] calcd. for $C_{23}H_{30}F_3N_3O_5P^-$, 516.1881; found 516.1881.

Synthesis and characterisation of diethyl {[7-(3-aminophenyl)-2,3-dioxo-6-(trifluoro methyl)-3,4-dihydroquinoxalin-1(2*H*)-yl]methyl}phosphonate (12)

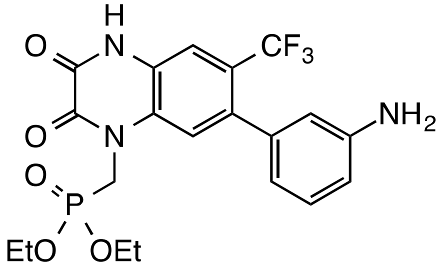

To a solution of compound **9** (932 mg, 1.80 mmol) in THF (90 mL) and Et_3N (1.28 g, 12.6 mmol, 1.76 mL) at r.t. was added ethyl chlorooxoacetate (**11**) (639 mg, 4.68 mmol, 0.52 mL) dropwise. The resulting mixture was stirred at r.t. for 20 h. After this time the reaction mixture was filtered and the filtrate was concentrated under reduced pressure. The resulting residue was dissolved in EtOH (60 mL) and a solution of HCl (60 mL, 1.0 M in H_2O) was added. The mixture was heated to 120 °C for 2.5 h, cooled to r.t. and the ethanol was removed under reduced pressure. The resulting aqueous layer was washed with CH_2Cl_2 (2 × 50 mL) and its pH was adjusted to 10-11 using a solution of Na_2CO_3 (2.0 M in H_2O). The aqueous layer was then back extracted with CH_2Cl_2 (2 × 50 mL). The combined organic extracts were dried over Na_2SO_4, filtered and concentrated under reduced pressure. Purification by flash column chromatography on silica gel eluting with CH_2Cl_2/MeOH (19:1) afforded compound **12** (505 mg, 60%) as an off-white solid.

mp: 305 – 308 °C, decomp; **TLC** (CH_2Cl_2/MeOH, 19:1): R_f = 0.29 (UV/ninhydrin); **^{1}H NMR** (400 MHz, CD_3OD): δ 7.57 (s, 1H), 7.44 (s, 1H), 7.13 (t, *J* = 8.0 Hz, 1H), 6.80 – 6.69 (m, 2H), 6.65 (d, *J* = 7.5 Hz, 1H), 4.80 (d, *J* = 12.5 Hz, 2H), 4.14 (dq, *J* = 7.0, 1.0 Hz, 4H), 1.23 (t, *J* = 7.0, 6H); **^{13}C NMR** (100 MHz, CD_3OD): δ 157.4 (d, *J* = 1.5 Hz), 155.2, 148.6, 141.0, 138.7 (q, *J* = 1.5 Hz), 129.6, 126.0, 125.3 (q, *J* = 31.0 Hz), 125.1 (q, *J* = 273.0 Hz), 120.8, 120.0 (q, *J* = 1.5 Hz), 117.2 (q, *J* = 1.5 Hz), 116.0, 115.1 (q, *J* = 5.5 Hz), 64.6 (d, *J* = 6.5 Hz), 39.9 (d, *J* = 157.0 Hz), 16.5 (d, *J* = 6.0 Hz); **^{19}F NMR** (376 MHz, CD_3OD): δ −57.9; **^{31}P NMR** (162 MHz, CD_3OD): δ 20.1; **IR** (neat): 3851, 3744, 3648, 3334, 2980, 1699, 1652, 1558, 1489, 1373, 1231, 1163, 1122, 1080, 1019, 787 cm^{-1}; **HRMS** (ESI, *m/z*): [$(M+H)^+$] calcd. for $C_{20}H_{22}F_3N_3O_5P^+$, 472.1244; found 472.1242.

Synthesis and characterisation of diethyl {[7-(3-{(*E*)-[4-(dimethylamino)phenyl]diazenyl} phenyl)-2,3-dioxo-6-(trifluoromethyl)-3,4-dihydroquinoxalin-1(2*H*)-yl]methyl}phosphonate (15)

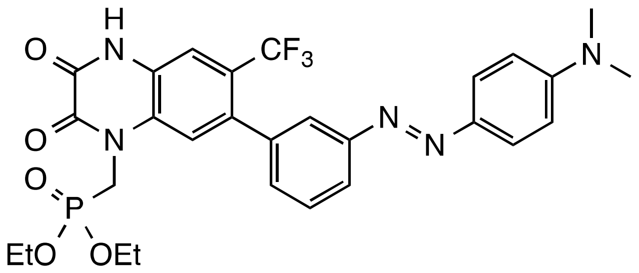

To a solution of compound **12** (95.0 mg, 0.200 mmol) in MeOH (4 mL) at 0 °C was added conc. HCl (0.1 mL) dropwise. *t*-Butyl nitrite (45.0 mg, 0.440 mmol, 52 µL, 90% purity) was added dropwise and the resulting solution was stirred at 0 °C for 1 h. The diazonium salt was then added dropwise to a flask containing *N*,*N*-dimethylaniline (**14**) (27.0 mg, 0.220 mmol, 28 µL) in MeOH (4 mL) and conc. HCl (0.1 mL) at 0 °C. The resulting solution was stirred at 0 °C for 1.5 h and then at r.t. for 2 h. The reaction was quenched with sat. aq. $NaHCO_3$ (20 mL) and extracted with EtOAc (2 × 20 mL). The combined organic extracts were dried over Na_2SO_4, filtered and concentrated under reduced pressure. Purification by flash column chromatography on silica gel eluting with CH_2Cl_2/MeOH (94:6) afforded compound **15** (106 mg, 88%) as an orange oil.

TLC (CH_2Cl_2/MeOH, 94:6): R_f = 0.31 (UV/ninhydrin); ^{1}**H NMR** (400 MHz, $CDCl_3$): δ 7.88 – 7.77 (m, 3H), 7.70 – 7.61 (m, 1H), 7.47 (t, *J* = 7.5 Hz, 1H), 7.41 – 7.29 (m, 3H), 6.76 – 6.69 (m, 2H), 4.70 – 4.57 (m, 2H), 4.24 – 4.10 (m, 4H), 3.07 (s, 6H), 1.27 – 1.18 (m, 6H); ^{13}**C NMR** (200 MHz, $CDCl_3$): δ 155.6 – 155.1 (m), 153.8 – 153.3 (m), 152.8, 152.7, 143.6, 139.5, 136.9, 130.1, 129.4 – 129.0 (m), 128.6, 128.1, 128.0, 125.2, 123.5 (q, *J* = 274.0 Hz), 123.0, 122.0, 119.3, 115.3 – 114.7 (m), 111.6, 63.6 (d, *J* = 6.0 Hz), 40.4, 39.4 (d, *J* = 156.5 Hz), 16.4 (d, *J* = 7.0 Hz); ^{19}**F NMR** (282 MHz, $CDCl_3$): δ −56.6; ^{31}**P NMR** (162 MHz, $CDCl_3$): δ 19.2; **IR** (thin film): 3058, 2983, 2905, 1698, 1622, 1598, 1518, 1364, 1264, 1235, 1153, 1120, 1047, 1017, 945, 822, 733, 700 cm^{-1}; **HRMS** (ESI, *m/z*): [$(M+H)^+$] calcd. for $C_{28}H_{30}F_3N_5O_5P^+$, 604.1931; found 604.1930.

Synthesis and characterisation of {[7-(3-{(*E*)-[4-(dimethylamino)phenyl]diazenyl}phenyl)-2,3-dioxo-6-(trifluoromethyl)-3,4-dihydroquinoxalin-1(2*H*)-yl]methyl}phosphonic acid TFA salt (ShuBQX-1)

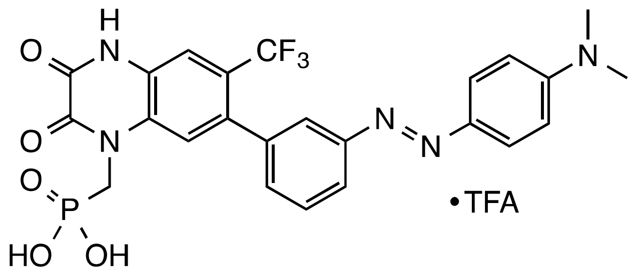

To a suspension of compound **15** (36.0 mg, 0.0600 mmol) in H_2O (1.5 mL) at r.t. was added conc. HCl (1.5 mL) dropwise. The resulting mixture was heated to 120 °C for 1.5 h and then cooled to r.t.. The reaction mixture was directly purified by reverse phase column chromatography eluting with H_2O [0.1% TFA]/MeCN [0.1% TFA] (9:1 $\rightarrow$ 7:3). The H_2O was removed by lyophilisation to afford compound **ShuBQX-1** (21 mg, 52%) as a dark red powder.

mp: >350 °C; ^{1}**H NMR** (800 MHz, $(CD_3)_2SO$): δ 12.35 (s, 1H), 7.83 (ddd, *J* = 8.0, 2.0, 1.0 Hz, 1H), 7.81 – 7.78 (m, 2H), 7.76 (t, *J* = 2.0 Hz, 1H), 7.64 (s, 1H), 7.61 (s, 1H), 7.60 (t, *J* = 8.0 Hz, 1H), 7.41 – 7.39 (m, 1H), 6.86 – 6.81 (m, 2H), 4.56 (d, *J* = 12.0 Hz, 2H), 3.06 (s, 6H); ^{13}**C NMR** (200 MHz, $(CD_3)_2SO$): δ 171.5, 158.1 (q, *J* = 35.0 Hz), 155.0, 153.2, 152.7, 152.0, 142.5, 139.7, 134.1, 130.1, 129.0 – 128.8 (m), 125.1, 124.9, 123.9 (q, *J* = 273.5 Hz), 122.7, 121.6 (q, *J* = 31.5 Hz), 121.0, 120.5, 113.1 (q, *J* = 6.0 Hz), 111.6, 40.0 (d, *J* = 156.0 Hz, estimated from HSQC), 39.6 (estimated from HSQC); note that only one CF_3 peak was observed in this spectrum; ^{19}**F NMR** (376 MHz, $(CD_3)_2SO$): δ −54.8, −74.5; ^{31}**P NMR** (162 MHz, CD_3OD): δ 14.0; **IR** (neat): 3070, 2892, 1697, 1608, 1559, 1374, 1276, 1241, 1159, 1132, 1079, 932, 850, 828, 799, 714 cm^{-1}; **HRMS** (ESI, *m/z*): [$(M+H)^+$] calcd. for $C_{24}H_{22}F_3N_5O_5P^+$, 548.1305; found 548.1311; **LCMS**: H_2O [0.1% FA]/MeCN [0.1% FA] (90:10 $\rightarrow$ 5:95), flow rate 1.0 mL/min over 8 min; $\mathbf{t_R}$ = 6.062 min, **MS** (ESI, *m/z*): [$(M+H)^+$] = 548.0, **UV-Vis**: λ_{max} = 430 nm.

Synthesis and characterisation of diethyl [({4'-[(*tert*-butoxycarbonyl)amino]-4-nitro-6-(trifluoromethyl)biphenyl-3-yl}amino)methyl]phosphonate (8)

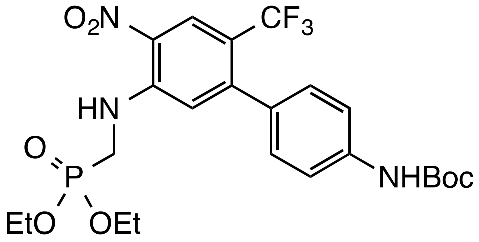

To a mixture of compound **4** (254 mg, 0.650 mmol) and 4-(*N*-boc-amino)phenylboronic acid (**6**) (170 mg, 0.715 mmol) in PhMe/EtOH (3.5:1, 15.5 mL) at r.t. under argon was added a solution of Na_2CO_3 (1.5 mL, 2.0 M in H_2O) and $Pd(PPh_3)_4$ (38.0 mg, 0.0330 mmol). The resulting mixture was degassed and flushed with argon (× 3) and then heated to 95 °C for 16 h. The reaction mixture was cooled to r.t., concentrated under reduced pressure and the resulting residue was directly purified by flash column chromatography eluting with CH_2Cl_2/EtOAc (4:1) to afford compound **8** (273 mg, 77%) as a yellow oil.

TLC (CH_2Cl_2/EtOAc, 4:1): R_f = 0.22 (UV/ninhydrin); **^{1}H NMR** (400 MHz, $CDCl_3$): δ 8.57 (s, 1H), 8.44 (q, *J* = 6.0 Hz, 1H), 7.47 – 7.40 (m, 2H), 7.29 – 7.22 (m, 2H), 6.82 (s, 1H), 6.71 (s, 1H), 4.17 (dq, *J* = 8.5, 7.0 Hz, 4H), 3.70 (dd, *J* = 13.5, 6.0 Hz, 2H), 1.52 (s, 9H), 1.32 (t, *J* = 7.0 Hz, 6H); **^{13}C NMR** (100 MHz, $CDCl_3$): δ 152.7, 148.6 (q, *J* = 1.0 Hz), 145.6 – 145.4 (m), 139.1, 132.7, 130.7, 129.2 (q, *J* = 2.0 Hz), 126.2 (q, *J* = 6.0 Hz), 123.5 (q, *J* = 272.5 Hz), 118.0, 117.7, 117.5 (q, *J* = 32.0 Hz), 81.0, 63.1 (d, *J* = 7.0 Hz), 39.4 (d, *J* = 157.5 Hz), 28.4, 16.6 (d, *J* = 5.5 Hz); **^{19}F NMR** (282 MHz, $CDCl_3$): δ −56.5; **^{31}P NMR** (162 MHz, CD_3OD): δ 20.6; **IR** (thin film): 3355, 3100, 2980, 2930, 1721, 1624, 1574, 1521, 1405, 1366, 1346, 1314, 1287, 1264, 1232, 1151, 1115, 1047, 1016, 971, 836, 764, 735 cm^{-1}; **HRMS** (ESI, *m/z*): [$(M+Na)^+$] calcd. for $C_{23}H_{29}F_3N_3NaO_7P^+$, 570.1587; found 570.1587.

Synthesis and characterisation of diethyl [({4-amino-4'-[(*tert*-butoxycarbonyl)amino]-6-(trifluoromethyl)biphenyl-3-yl}amino)methyl]phosphonate (10)

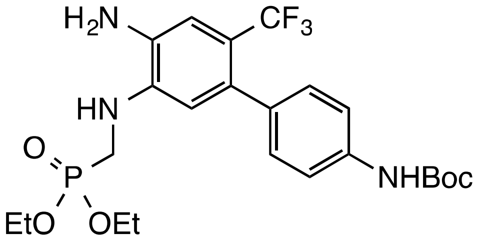

To a solution of compound **8** (328 mg, 0.600 mmol) in MeOH (12 mL) at r.t. was added Pd/C (33.0 mg, 10 wt. % Pd labelling). The reaction vessel was flushed with hydrogen (× 3) and the resulting mixture was stirred at r.t. for 16 h. The reaction mixture was filtered through a pad of Celite® washing with MeOH (20 mL) and the filtrate was concentrated under reduced pressure. Purification by flash column chromatography on silica gel eluting with EtOAc/MeOH (19:1 → 9:1) to afford compound **10** (168 mg, 54%) as a colourless oil.

TLC (EtOAc/MeOH, 19:1): R_f = 0.55 (UV/ninhydrin); **^{1}H NMR** (400 MHz, CD_3OD): δ 7.38 (d, *J* = 8.0 Hz, 2H), 7.18 (d, *J* = 8.0 Hz, 2H), 7.06 (s, 1H), 6.58 (s, 1H), 4.10 (q, *J* = 7.0 Hz, 4H), 3.67 (d, *J* = 10.5 Hz, 2H), 1.52 (s, 9H), 1.25 (t, *J* = 7.0 Hz, 6H); **^{13}C NMR** (100 MHz, CD_3OD): δ 155.3, 139.4, 139.4, 136.8, 134.7, 133.8 (q, *J* = 2.0 Hz), 130.7 (q, *J* = 1.0 Hz), 126.4 (q, *J* = 271.5 Hz), 118.9, 118.8 (q, *J* = 30.0 Hz), 115.5, 114.3 (q, *J* = 5.5 Hz), 80.8, 64.1 (d, *J* = 7.0 Hz), 40.4 (d, *J* = 159.0 Hz), 28.7, 16.7 (d, *J* = 6.0 Hz); **^{19}F NMR** (376 MHz, CD_3OD): δ −56.2; **^{31}P NMR** (162 MHz, CD_3OD): δ 24.8; **IR** (neat): 3364, 2978, 2930, 1703, 1584, 1511, 1431, 1403, 1391, 1366, 1312, 1232, 1150, 1103, 1048, 1017, 971, 897, 838, 805, 773 cm^{-1}; **HRMS** (ESI, *m/z*): [$(M+Na)^+$] calcd. for $C_{23}H_{31}F_3N_3NaO_5P^+$, 540.1846; found 540.1844.

Synthesis and characterisation of diethyl {[7-(4-aminophenyl)-2,3-dioxo-6-(trifluoro methyl)-3,4-dihydroquinoxalin-1(2*H*)-yl]methyl}phosphonate (13)

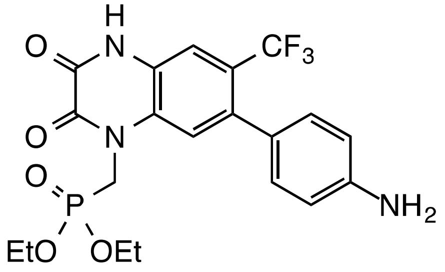

To a solution of compound **10** (124 mg, 0.240 mmol) in THF (12 mL) and Et_3N (170 mg, 1.68 mmol, 234 μL) at r.t. was added ethyl chlorooxoacetate (**11**) (85.0 mg, 0.624 mmol, 69 μL) dropwise. The resulting mixture was stirred at r.t. for 20 h. After this time the reaction mixture was filtered and the filtrate was concentrated under reduced pressure. The resulting residue was dissolved in EtOH (8 mL) and a solution of HCl (8 mL, 1.0 M in H_2O) was added. The mixture was heated to 120 °C for 2.5 h, cooled to r.t. and the ethanol was removed under reduced pressure. The resulting aqueous layer was washed with CH_2Cl_2 (2 × 20 mL) and its pH was adjusted to 10-11 using a solution of Na_2CO_3 (2.0 M in H_2O). The aqueous layer was then back extracted with CH_2Cl_2 (2 × 20 mL). The combined organic extracts were dried over Na_2SO_4, filtered and concentrated under reduced pressure. Purification by flash column chromatography on silica gel eluting with CH_2Cl_2/MeOH (19:1) afforded compound **13** (62 mg, 55%) as an off-white solid.

mp: 315 – 318 °C, decomp; **TLC** (CH_2Cl_2/MeOH, 19:1): R_f = 0.27 (UV/ninhydrin); **^{1}H NMR** (400 MHz, CD_3OD): δ 7.57 (s, 1H), 7.42 (s, 1H), 7.15 – 7.08 (m, 2H), 6.79 – 6.73 (m, 2H), 4.82 (d, *J* = 12.0 Hz, 2H), 4.19 – 4.08 (m, 4H), 1.23 (t, *J* = 7.0 Hz, 6H); **^{13}C NMR** (100 MHz, CD_3OD): δ 157.8, 156.0 – 155.7 (m), 149.1, 138.8 (q, *J* = 2.0 Hz), 131.0 (q, *J* = 2.0 Hz), 129.7, 129.5, 126.5 – 126.3 (m), 125.6 (q, *J* = 30.5 Hz), 125.3 (q, *J* = 273.0 Hz), 121.1, 116.1 – 115.8 (m), 115.7, 64.6 (d, *J* = 6.5 Hz), 39.9 (d, *J* = 156.5 Hz), 16.6 (d, *J* = 6.0 Hz); **^{19}F NMR** (376 MHz, CD_3OD): δ −57.9; **^{31}P NMR** (162 MHz, CD_3OD): δ 20.2; **IR** (neat): 3360, 2984, 1698, 1620, 1505, 1381, 1350, 1238, 1182, 1158, 1119, 1080, 1019, 835 cm^{-1}; **HRMS** (ESI, *m/z*): [$(M+H)^+$] calcd. for $C_{20}H_{22}F_3N_3O_5P^+$, 472.1244; found 472.1240.

Synthesis and characterisation of diethyl {[7-(4-{(*E*)-[4-(dimethylamino)phenyl]diazenyl}phenyl)-2,3-dioxo-6-(trifluoromethyl)-3,4-dihydroquinoxalin-1(2*H*)-yl]methyl}phosphonate (16)

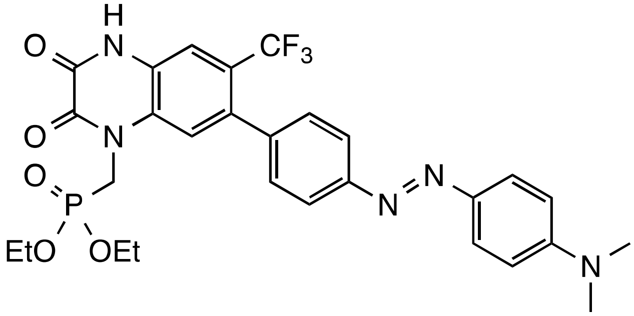

To a solution of compound **13** (38.0 mg, 0.0800 mmol) in MeOH (1.6 mL) at 0 °C was added conc. HCl (0.04 mL) dropwise. *t*-Butyl nitrite (17.0 mg, 0.160 mmol, 21 µL, 90% purity) was added dropwise and the resulting solution was stirred at 0 °C for 30 min. The diazonium salt was then added dropwise to a flask containing *N*,*N*-dimethylaniline (**14**) (11.0 mg, 0.0880 mmol, 11 µL) in MeOH (1.6 mL) and conc. HCl (0.04 mL) at 0 °C. The resulting solution was stirred at 0 °C for 2 h and then at r.t. for 2 h. The reaction was quenched with sat. aq. $NaHCO_3$ (10 mL) and extracted with EtOAc (2 × 10 mL). The combined organic extracts were dried over Na_2SO_4, filtered and concentrated under reduced pressure. Purification by flash column chromatography on silica gel eluting with CH_2Cl_2/MeOH (98:2 → 95:5) afforded compound **16** (28 mg, 58%) as an orange solid.

mp: 215 – 218 °C; **TLC** (CH_2Cl_2/MeOH, 19:1): R_f = 0.36 (UV/CAM); **^{1}H NMR** (400 MHz, $CDCl_3$): δ 7.91 – 7.84 (m, 2H), 7.83 – 7.75 (m, 2H), 7.61 (s, 1H), 7.47 – 7.40 (m, 2H), 7.36 (s, 1H), 6.79 – 6.70 (m, 2H), 4.69 (d, *J* = 12.0 Hz, 2H), 4.30 – 4.13 (m, 4H), 3.09 (s, 6H), 1.26 (t, *J* = 7.0 Hz, 6H); **^{13}C NMR** (100 MHz, $CDCl_3$): δ 155.2, 153.5, 152.8, 152.7, 143.7, 139.7, 136.8 (q, *J* = 1.5 Hz), 130.0, 128.1, 125.3, 125.0 (q, *J* = 31.5 Hz), 124.3, 123.5 (q, *J* = 273.5 Hz), 121.8, 119.3, 114.9 (q, *J* = 5.0 Hz), 111.7, 63.7 (d, *J* = 6.5 Hz), 40.4, 39.4 (d, *J* = 156.0 Hz), 16.4 (d, *J* = 6.0 Hz), **^{19}F NMR** (376 MHz, $CDCl_3$): δ −56.5; **^{31}P NMR** (162 MHz, $CDCl_3$): δ 19.2; **IR** (thin film): 3153, 3075, 2984, 2909, 1704, 1622, 1600, 1519, 1421, 1366, 1312, 1237, 1157, 1139, 1082, 1047, 1020, 846, 822 cm^{-1}; **HRMS** (ESI, *m/z*): [$(M+H)^+$] calcd. for $C_{28}H_{30}F_3N_5O_5P^+$, 604.1931; found 604.1941.

Synthesis and characterisation of {[7-(4-{(*E*)-[4-(dimethylamino)phenyl]diazenyl}phenyl)-2,3-dioxo-6-(trifluoromethyl)-3,4-dihydroquinoxalin-1(2*H*)-yl]methyl}phosphonic acid TFA salt (ShuBQX-2)

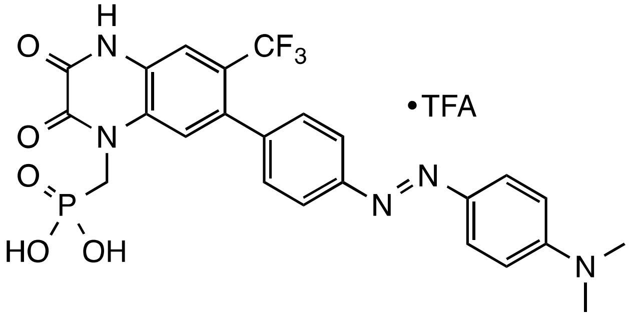

To a suspension of compound **16** (24.0 mg, 0.0400 mmol) in H_2O (1 mL) at r.t. was added conc. HCl (1 mL) dropwise. The resulting mixture was heated to 120 °C for 12 h and then cooled to r.t.. The reaction mixture was directly purified by reverse phase column chromatography eluting with H_2O [0.1% TFA]/MeCN [0.1% TFA] (9:1 $\rightarrow$ 7:3). The H_2O was removed by lyophilisation to afford compound **ShuBQX-2** (5.6 mg, 21%) as a dark brown solid.

mp: >350 °C; **^{1}H NMR** (800 MHz, $(CD_3)_2SO$): δ 12.33 (s, 1H), 7.86 – 7.77 (m, 4H), 7.65 (s, 1H), 7.59 (s, 1H), 7.52 – 7.46 (m, 2H), 6.89 – 6.81 (m, 2H), 4.49 (br s, 2H), 3.07 (s, 6H); **^{13}C NMR** (200 MHz, $(CD_3)_2SO$): δ 157.7, 157.6, 154.9, 153.2, 152.7, 151.9, 142.6, 139.8, 134.0, 130.1, 128.9, 125.0, 124.9, 123.9 (q, *J* = 274.0 Hz), 121.3, 120.6, 113.1 (q, *J* = 5.0 Hz), 111.6, 40.0 (d, *J* = 155.5 Hz, estimated by HSQC), 39.6 (estimated by HSQC); note that only one CF_3 peak was observed in this spectrum; **^{19}F NMR** (376 MHz, $(CD_3)_2SO$): δ −54.7, −73.4; **^{31}P NMR** (162 MHz, $(CD_3)_2SO$): δ 13.4; **IR** (neat): 3063, 2950, 1682, 1671, 1620, 1594, 1543, 1367, 1350, 1274, 1241, 1207, 1169, 1137, 1123, 1108, 1074, 955, 937, 909, 894, 838, 824, 726 cm^{-1}; **HRMS** (ESI, *m/z*): $[(M+H)^+]$ calcd. for $C_{24}H_{22}F_3N_5O_5P^+$, 548.1305; found 548.1212; **LCMS**: H_2O [0.1% FA]/MeCN [0.1% FA] (90:10 $\rightarrow$ 5:95), flow rate 1.0 mL/min over 8 min; $\mathbf{t_R}$ = 6.095 min, **MS** (ESI, *m/z*): $[(M+H)^+]$ = 548.0, **UV-Vis**: λ_{max} = 435 nm.

Synthesis and characterisation of *tert*-butyl 2-[5-{[(diethoxyphosphoryl)methyl]amino}-4-nitro-2-(trifluoromethyl)phenyl]-1-phenylhydrazinecarboxylate (18)

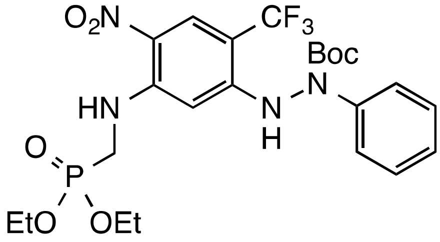

To a mixture of compound **4** (200 mg, 0.512 mmol) and hydrazide **17** (120 mg, 0.576 mmol) in PhMe (6 mL) at r.t. was added Cs_2CO_3 (280 mg, 0.726 mmol), $Pd(OAc)_2$ (44.0 mg, 0.196 mmol) and a solution of P(*t*-Bu)$_3$ (0.510 mL, 0.510 mmol, 1.0 M in PhMe). The resulting mixture was stirred for 30 min. at r.t. and then heated to 120 °C for 2 h. The reaction mixture was cooled to r.t., concentrated under reduced pressure and the resulting residue was directly purified by flash column chromatography eluting with *i*-Hex/EtOAc (8:2 $\rightarrow$ 1:1) to afford compound **18** (163 mg, 57%) as an orange solid.

mp: 230 °C, decomp; **TLC** (*i*-Hex/EtOAc, 1:1): R_f = 0.37 (UV/ninhydrin); ^{1}**H NMR** (400 MHz, CD_3OD): δ 8.40 (s, 1H), 7.64 – 7.55 (m, 2H), 7.42 – 7.33 (m, 2H), 7.24 – 7.14 (m, 1H), 6.30 (s, 1H), 4.12 – 3.97 (m, 4H), 3.71 (d, *J* = 12.0 Hz, 2H), 1.47 (s, 9H), 1.23 (t, *J* = 7.0 Hz, 6H); ^{13}**C NMR** (100 MHz, CD_3OD): δ 154.9, 151.7, 149.4 (d, *J* = 5.0 Hz), 143.0, 129.8, 128.6 (q, *J* = 6.0 Hz), 126.6, 125.9, 123.7, 104.6 (q, *J* = 32.5 Hz), 94.4, 83.8, 64.4 (d, *J* = 7.0 Hz), 39.7 (d, *J* = 158.0 Hz), 28.4, 16.7 (d, *J* = 5.5 Hz); note that the CF_3 peak was not observed in this spectrum; ^{19}**F NMR** (376 MHz, CD_3OD): δ −62.8; ^{31}**P NMR** (162 MHz, CD_3OD): δ 22.3; **IR** (thin film): 3346, 2981, 2931, 1723, 1633, 1577, 1541, 1425, 1355, 1339, 1301, 1251, 1213, 1153, 1112, 1048, 1022, 972, 761 cm^{-1}; **HRMS** (ESI, *m/z*): [(M−H)$^{-}$] calcd. for $C_{23}H_{30}F_3N_4NaO_7P^{-}$, 561.1731; found 561.1739.

Synthesis and characterisation of *tert*-butyl 2-[4-amino-5-{[(diethoxyphosphoryl)methyl] amino}-2-(trifluoromethyl)phenyl]-1-phenylhydrazinecarboxylate (19)

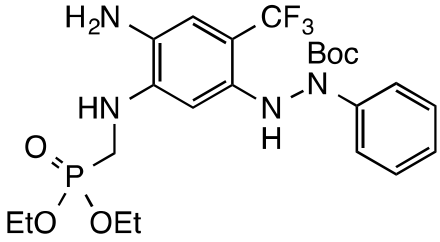

To a solution of compound **18** (163 mg, 0.290 mmol) in MeOH (5 mL) at r.t. was added Pd/C (34.0 mg, 20 wt. % Pd labelling). The reaction vessel was flushed with hydrogen (× 3) and the resulting mixture was stirred at r.t. for 5.5 h after which more Pd/C (16.0 mg, 10 wt. % Pd labelling) was added. The reaction mixture was stirred for 2.5 h at r.t. and then filtered through a pad of Celite® washing with EtOAc (20 mL). The filtrate was concentrated under reduced pressure to give a brown solid (132 mg, 86%), which was used in the next step without further purification. An analytical sample was obtained by purification using preparative TLC eluting with CH_2Cl_2/MeOH (19:1).

mp: 155 – 156 °C; **TLC** (CH_2Cl_2/MeOH, 19:1): R_f = 0.29 (UV/ninhydrin); **^{1}H NMR** (400 MHz, CD_3OD): δ 7.67 – 7.59 (m, 2H), 7.39 – 7.28 (m, 2H), 7.13 (t, *J* = 7.5 Hz, 1H), 6.94 (s, 1H), 6.25 (s, 1H), 4.08 – 3.92 (m, 4H), 3.51 (d, *J* = 10.5 Hz, 2H), 1.40 (s, 9H), 1.20 (t, *J* = 7.1 Hz, 6H); **^{13}C NMR** (100 MHz, CD_3OD): δ 155.9, 142.9 (d, *J* = 4.6 Hz), 142.1 – 141.9 (m), 129.4, 127.6, 126.8 (q, *J* = 270.0 Hz), 125.8, 123.5, 116.2 – 115.8 (m), 104.0 (q, *J* = 31.0 Hz), 97.1 (q, *J* = 2.0 Hz), 83.2, 64.4 – 63.7 (m), 41.5 – 41.3 (m, part of N**C**H_2PO multiplet), 39.9 – 39.7 (m, part of N**C**H_2PO multiplet), 28.7 – 28.0 (m), 17.0 – 16.3 (m); **^{19}F NMR** (376 MHz, CD_3OD): δ −60.4; **^{31}P NMR** (162 MHz, CD_3OD): δ 24.4; **IR** (thin film): 3370, 2981, 2931, 1713, 1622, 1597, 1537, 1489, 1453, 1392, 1369, 1335, 1300, 1245, 1198, 1150, 1094, 1048, 1023, 970, 867, 824, 757 cm^{-1}; **HRMS** (ESI, *m/z*): [$(M+Na)^+$] calcd. for $C_{23}H_{32}F_3N_4NaO_5P^+$, 555.1955; found 555.1959.

Synthesis and characterisation of diethyl (*E*)-((2,3-dioxo-7-(phenyldiazenyl)-6-(trifluoromethyl)-3,4-dihydroquinoxalin-1(2H)-yl)methyl)phosphonate (S1) and ({2,3-dioxo-7-[(*E*)-phenyldiazenyl]-6-(trifluoromethyl)-3,4-dihydroquinoxalin-1(2*H*)-yl}methyl)phosphonic acid (ShuBQX-3)

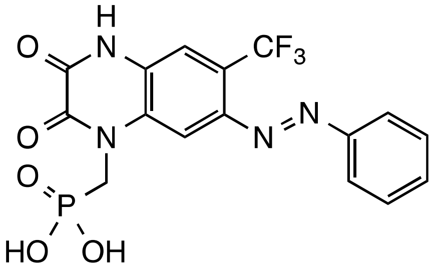

To a solution of compound **19** (132 mg, 0.248 mmol) in THF (11 mL) and Et_3N (174 mg, 1.72 mmol, 0.240 mL) at r.t. was added ethyl chlorooxoacetate (**11**) (88 mg, 0.644 mmol, 72 µL) dropwise. The resulting mixture was stirred at r.t. for 19 h. After this time the reaction mixture was filtered and the filtrate was concentrated under reduced pressure. The resulting residue was dissolved in EtOH (2 mL) and a solution of HCl (9 mL, 6.0 M in H_2O) was added. The resulting mixture was heated to 90 °C for 3 h and then cooled to r.t.. The reaction mixture was directly purified by reverse phase column chromatography eluting with H_2O [0.1% TFA]/MeCN [0.1% TFA] (9:1 → 6:4). The H_2O was removed by lyophilisation to afford the diethyl phosphonate of **ShuBQX-3** (**S1**) (6.90 mg, 6% over 2 steps) as an orange-red solid and **ShuBQX-3** (14.0 mg, 18% over three steps) as a dark brown solid.

Characterization data for ShuBQX-3:

mp: 275 – 280 °C, decomp; ^{1}**H NMR** (400 MHz, CD_3OD): δ 8.12 (s, 1H), 7.99 – 7.89 (m, 2H), 7.63 (s, 1H), 7.58 – 7.49 (m, 3H), 4.72 (d, *J* = 12.0 Hz, 2H); ^{13}**C NMR** (100 MHz, CD_3OD): δ 157.0, 155.3, 153.8, 145.8, 133.2, 131.1, 130.4, 129.0, 125.9 (q, *J* = 32.0 Hz), 124.8, q, *J* = 272.5 Hz), 124.4, 115.2, (q, *J* = 6.0 Hz), 105.5, 41.7 (d, *J* = 152.0 Hz); ^{19}**F NMR** (376 MHz, CD_3OD): δ −58.5; ^{31}**P NMR** (162 MHz, CD_3OD): δ 15.3; **IR** (thin film): 3415, 3073, 2961, 1694, 1619, 1393, 1358, 1298, 1269, 1241, 1136, 1062, 1019, 933, 771, 732, 687 cm^{-1}; **HRMS** (ESI, *m/z*): [$(M+H)^+$] calcd. for $C_{16}H_{13}F_3N_4O_5P^+$, 429.0570; found 429.0580; **LCMS**: H_2O [0.1% FA]/MeCN [0.1% FA] (90:10 → 10:90), flow rate 2.0 mL/min over 5 min; $\mathbf{t_R}$ = 2.858 min, **MS** (ESI, *m/z*): [$(M+H)^+$] = 429.0, **UV-Vis**: λ_{max} = 360 nm.

Characterization data for S1:

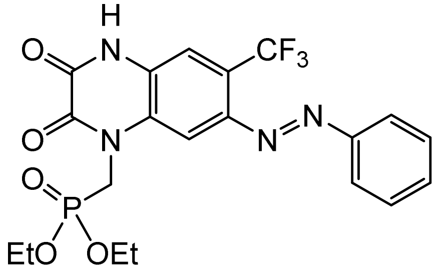

mp: 124 – 126 °C, **TLC** (CH_2Cl_2/MeOH, 18:2): R_f = 0.30 (UV/CAM); **^{1}H NMR** (400 MHz, CD_3OD): δ 8.08 (s, 1H), 7.98 – 7.95 (m, 2H), 7.71 (s, 1H), 7.59 (m, *J* = 5.4, 2.0 Hz, 3H), 4.26 – 4.17 (m, 4H), 1.30 (t, *J* = 7.1 Hz, 7H); **^{13}C NMR** (100 MHz, CD_3OD): δ 157.2, 155.1, 153.8, 145.8, 133.4, 130.9, 130.5, 129.2, 126.1, 124.4, 121.8, 115.5, 105.0, 64.8, 64.7, 40.04 (d, *J* = 156.9 Hz), 39.3, 16.7, 16.7; note that the CF_3 peak was not observed in this spectrum; **^{19}F NMR** (376 MHz, CD_3OD): δ −58.6; **^{31}P NMR** (162 MHz, CD_3OD): δ 20.2; **IR** (thin film): 3460, 2988, 1702, 1619, 1508, 1387, 1297, 1269, 1241, 1207, 1137, 1048, 1022, 981, 772 cm^{-1}; **HRMS** (ESI, *m/z*): [$(M+NH_4)^+$] calcd. for $C_{20}H_{24}F_3N_5O_5P^+$, 502.14617; found 502.14612.

NMR Spectra

^{1}H NMR spectrum of compound **3**

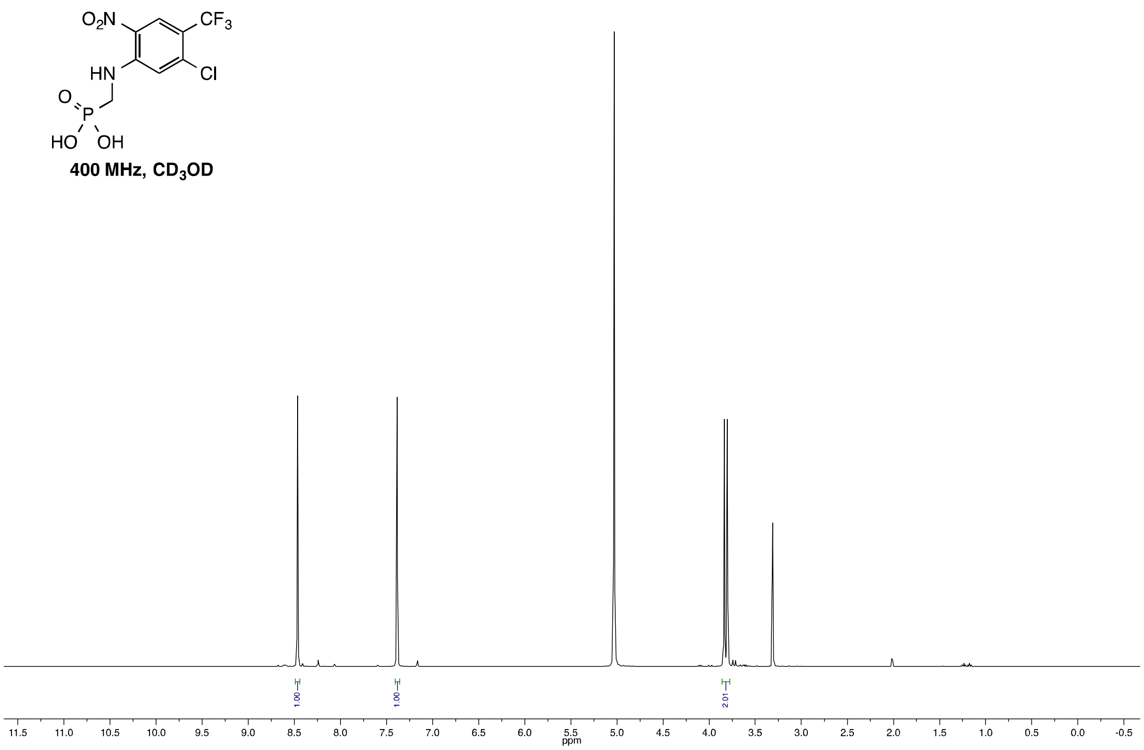

^{13}C NMR spectrum of compound **3**

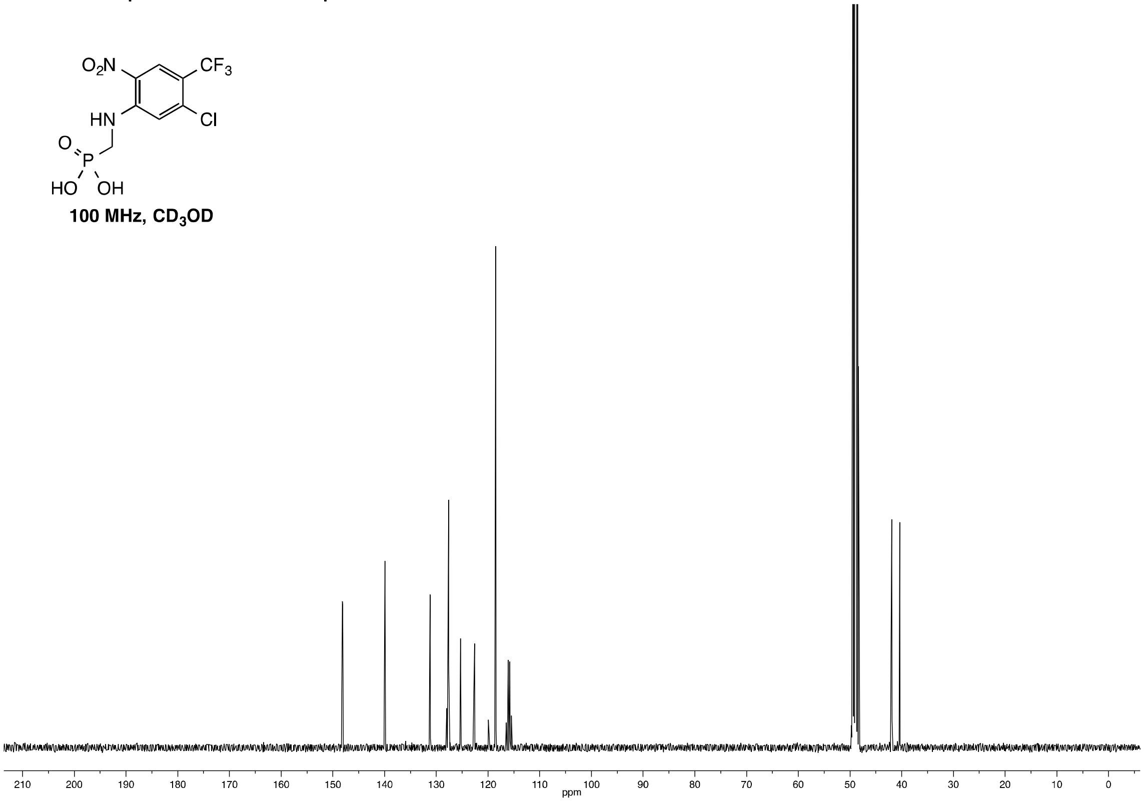

^{1}H NMR spectrum of compound **4**

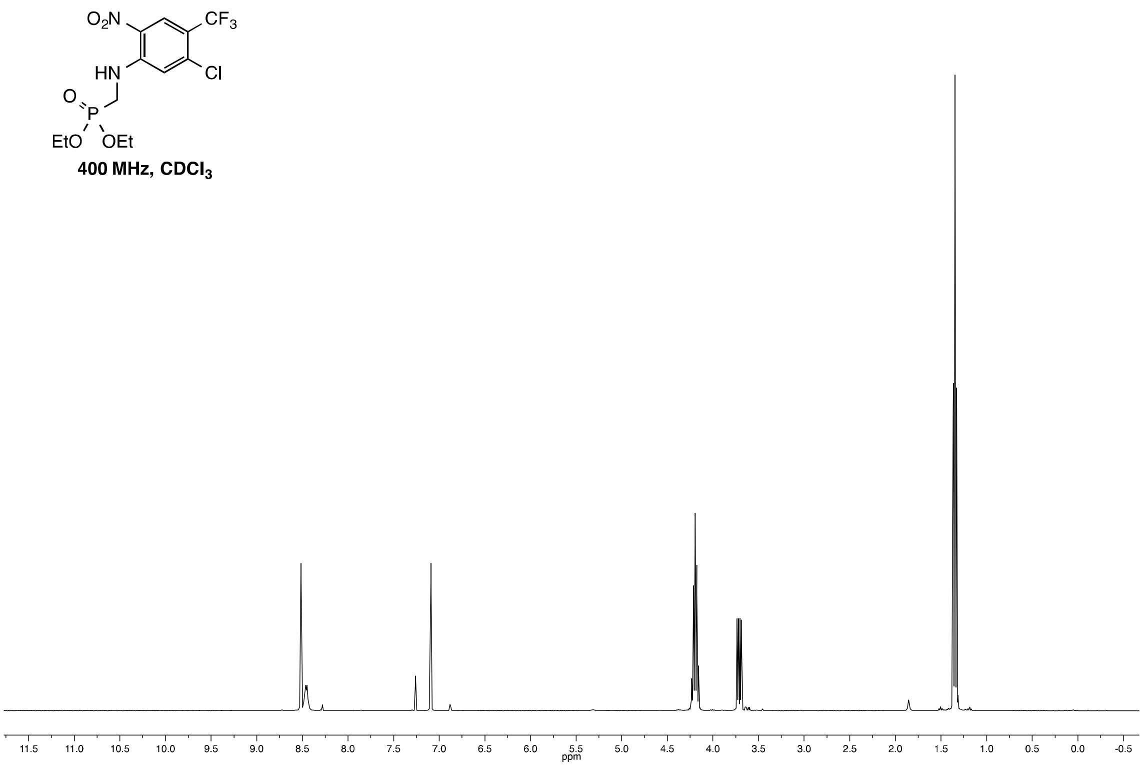

^{13}C NMR spectrum of compound **4**

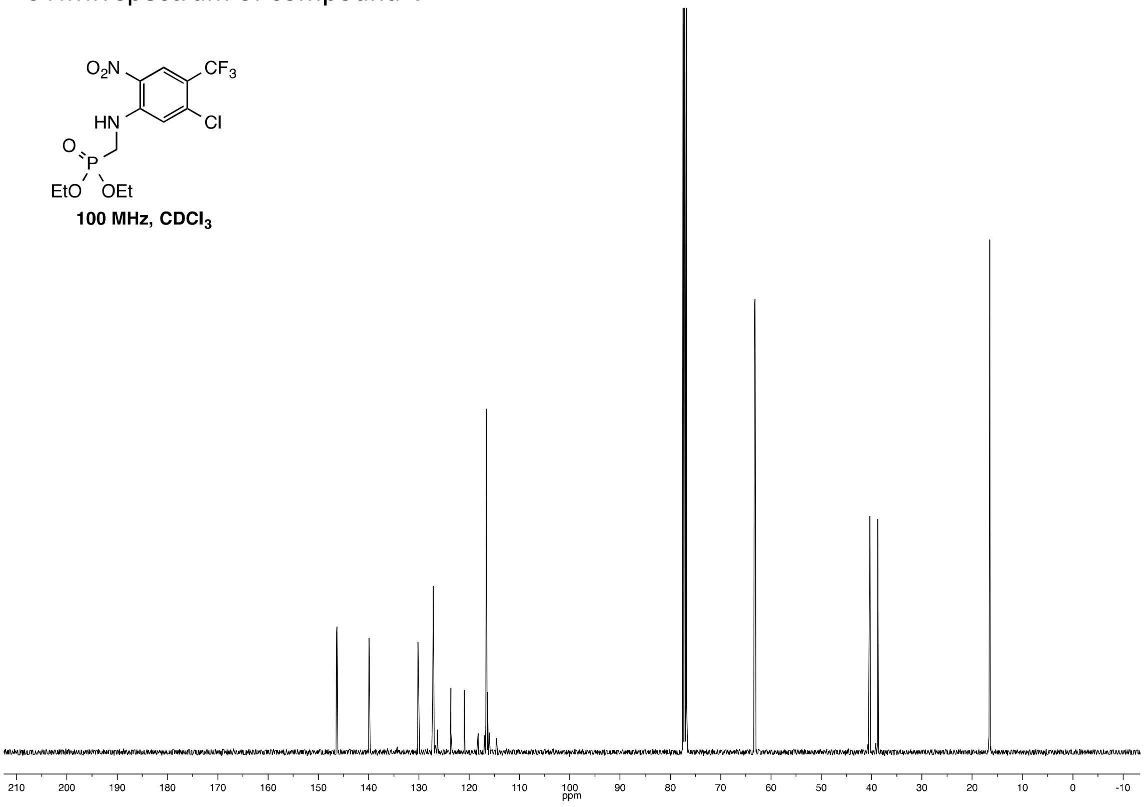

^{1}H NMR spectrum of compound **7**

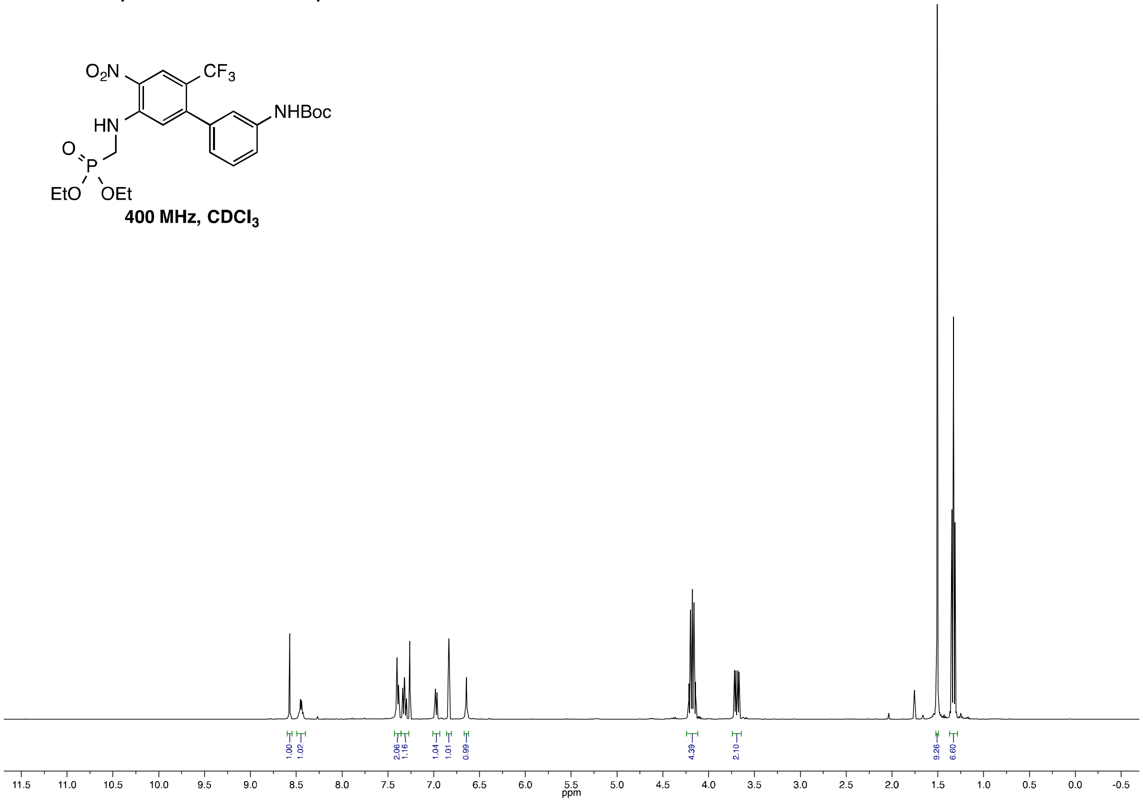

^{13}C NMR spectrum of compound **7**

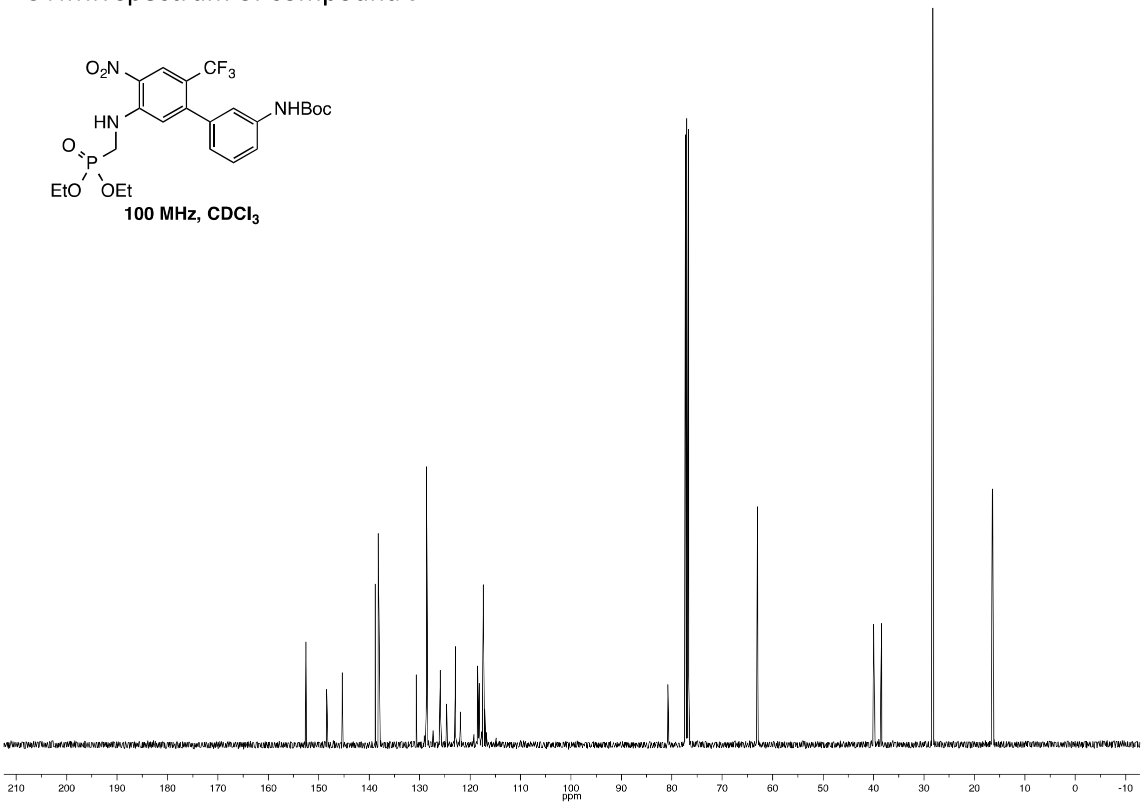

^{1}H NMR spectrum of compound **9**

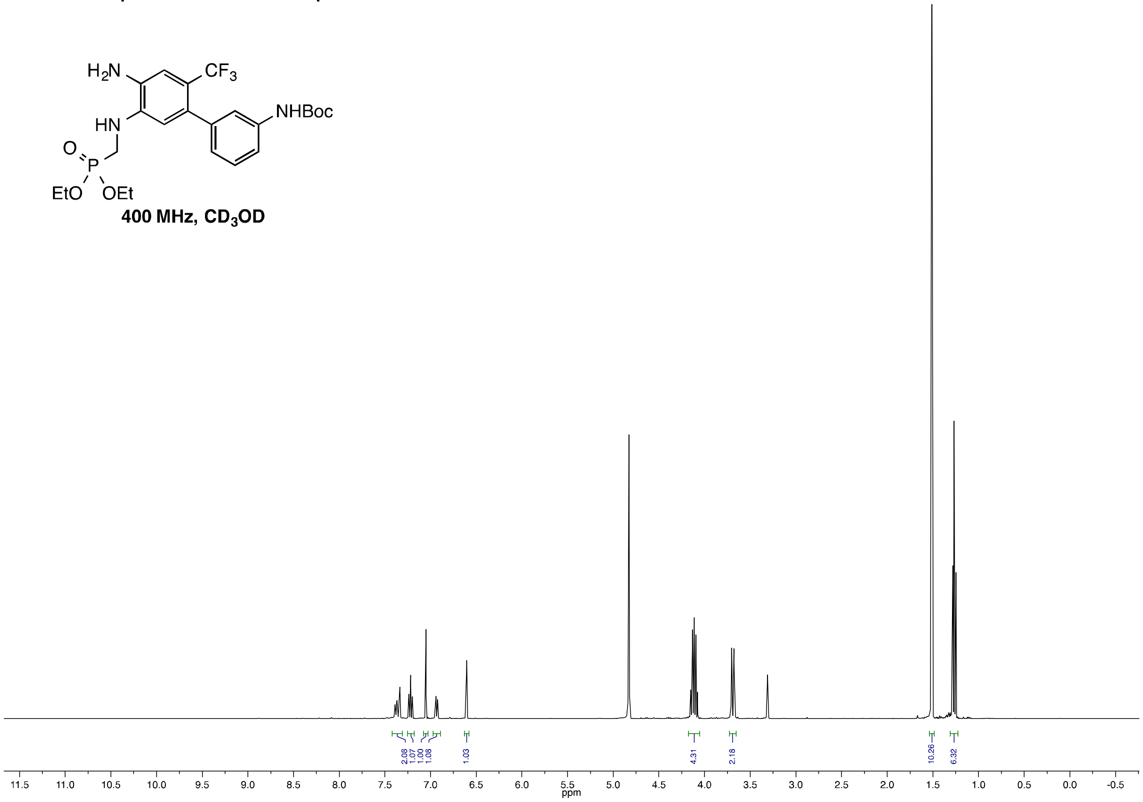

^{13}C NMR spectrum of compound **9**

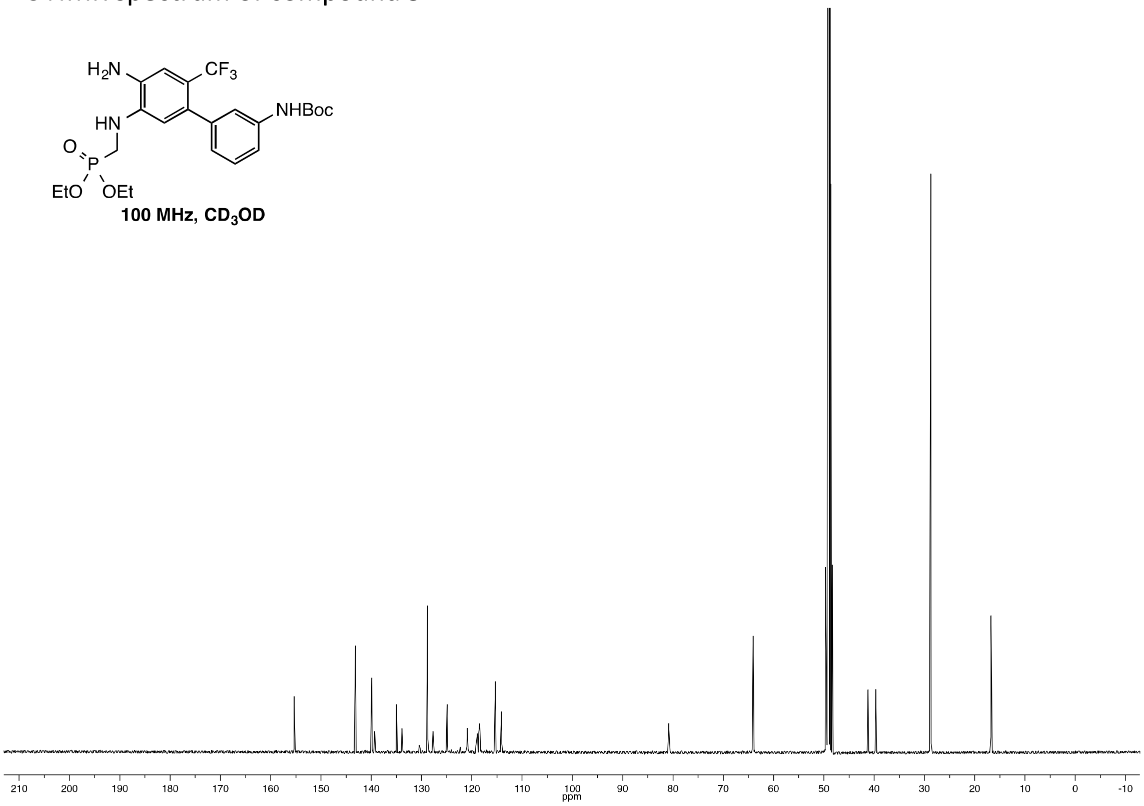

^{1}H NMR spectrum of compound **12**

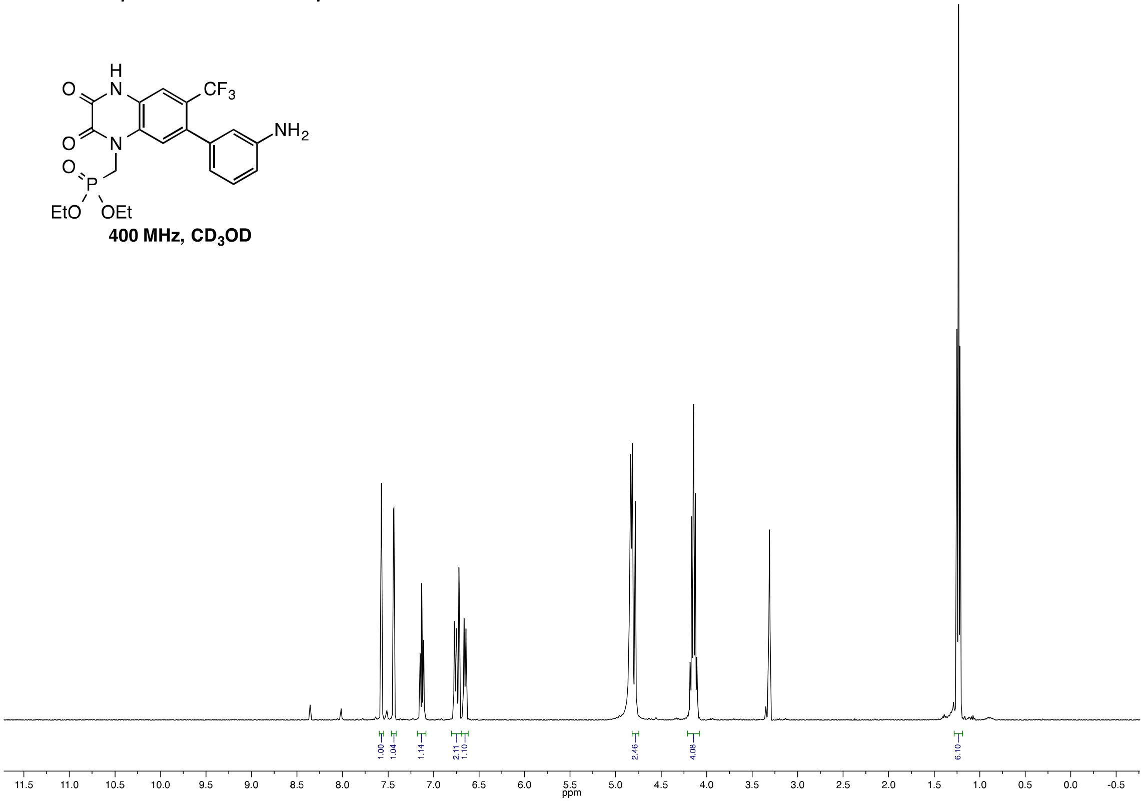

^{13}C NMR spectrum of compound **12**

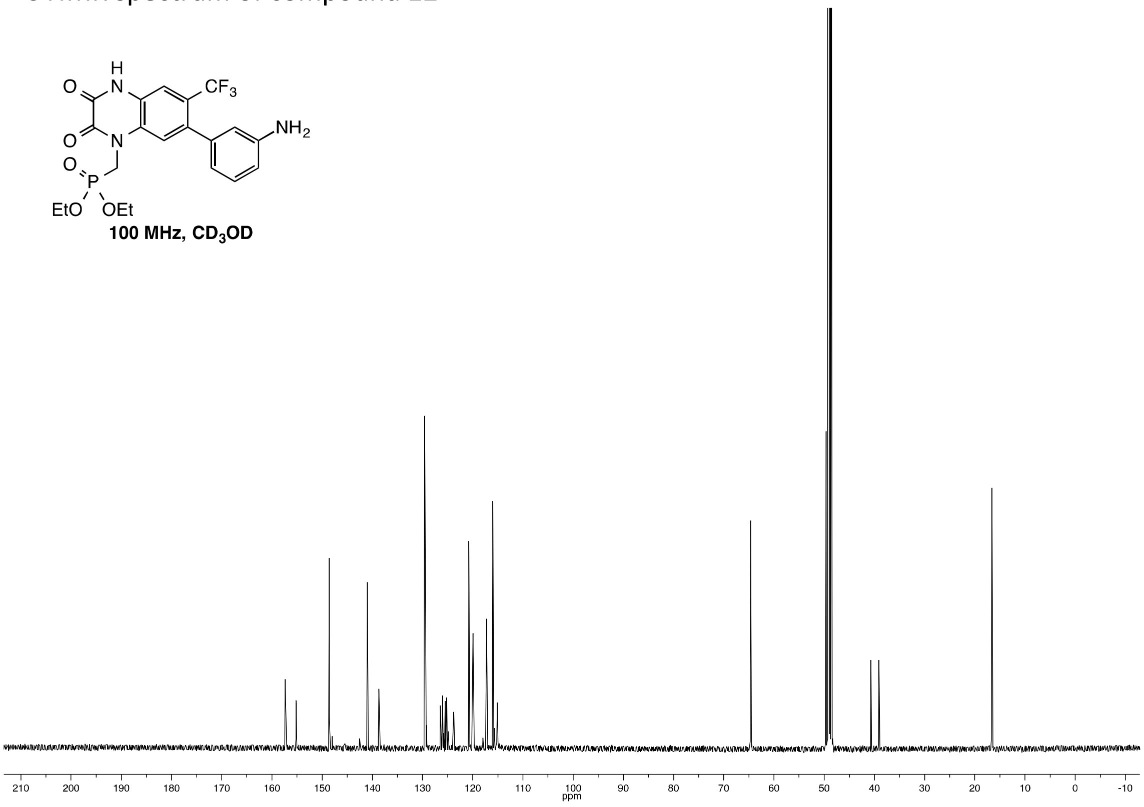

^{1}H NMR spectrum of compound **15**

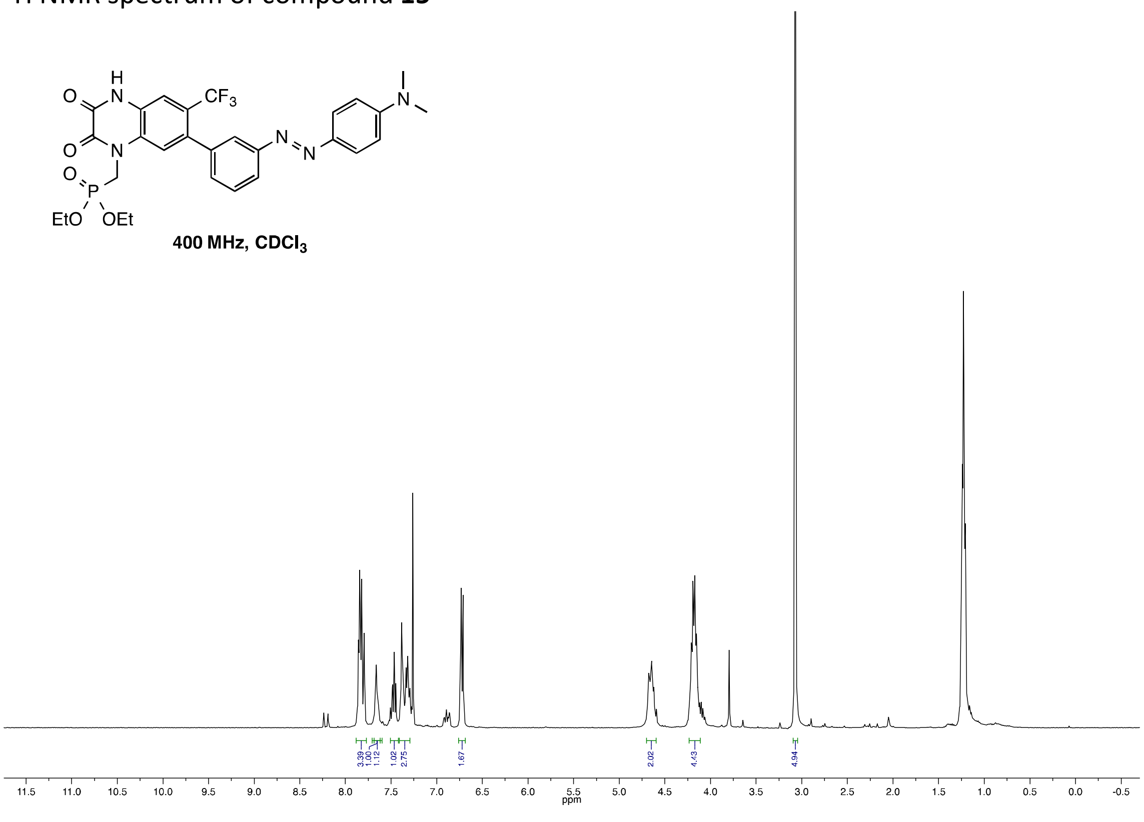

^{13}C NMR spectrum of compound **15**

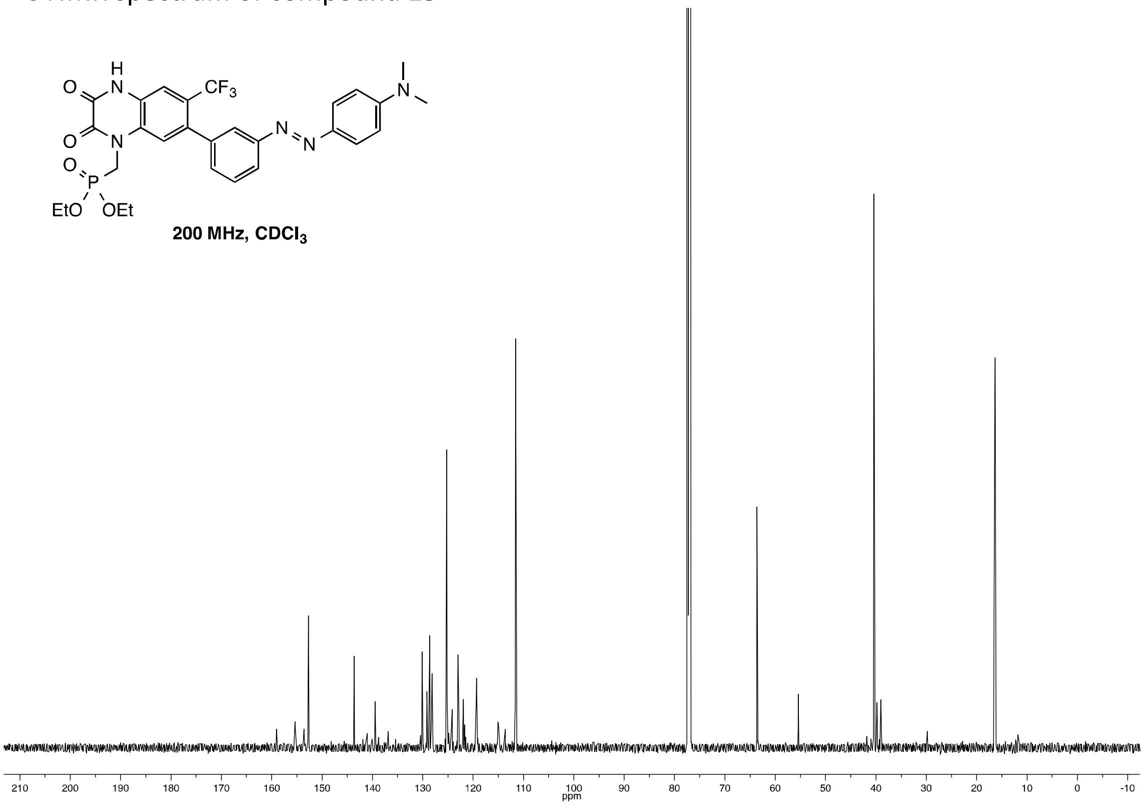

^{1}H NMR spectrum of compound **ShuBQX-1**

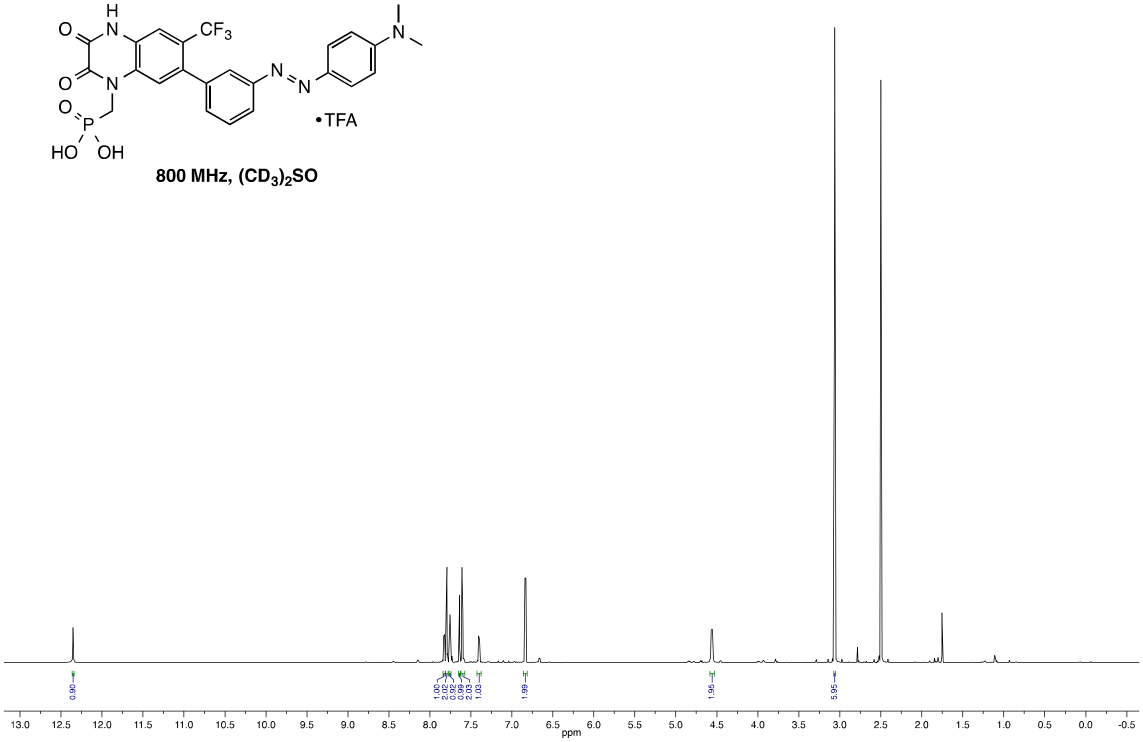

^{13}C NMR spectrum of compound **ShuBQX-1**

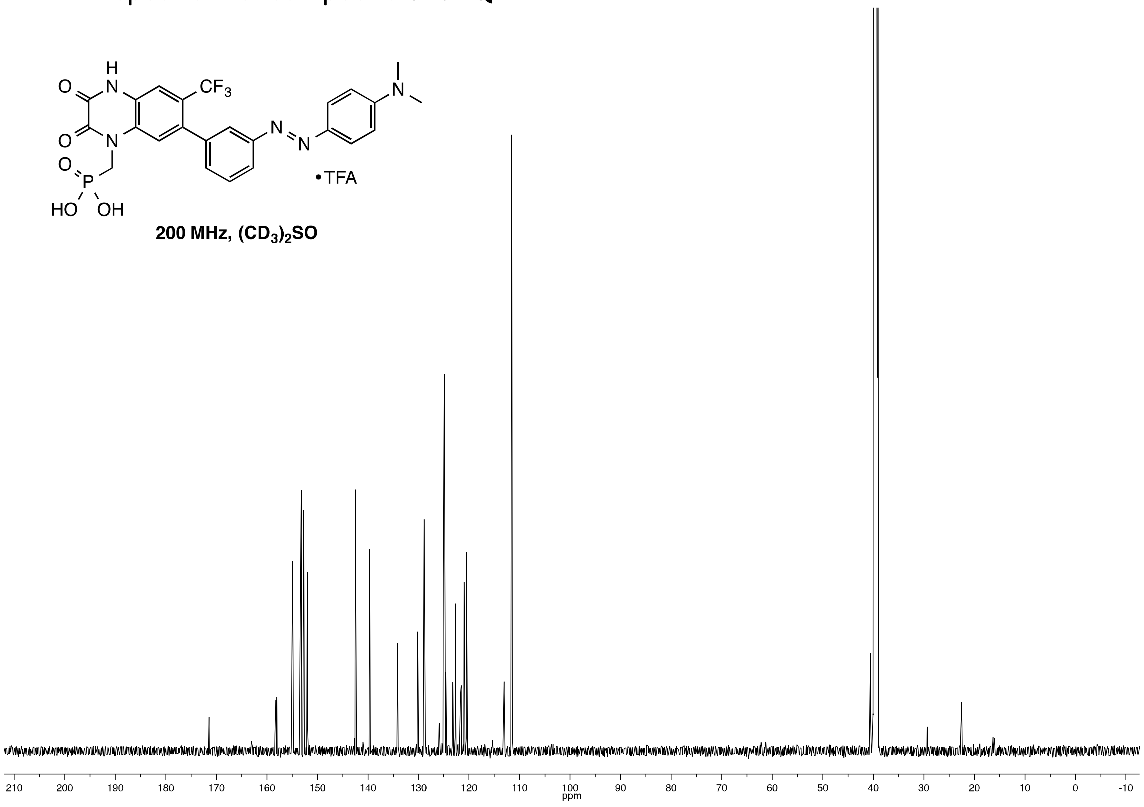

^{1}H NMR spectrum of compound **8**

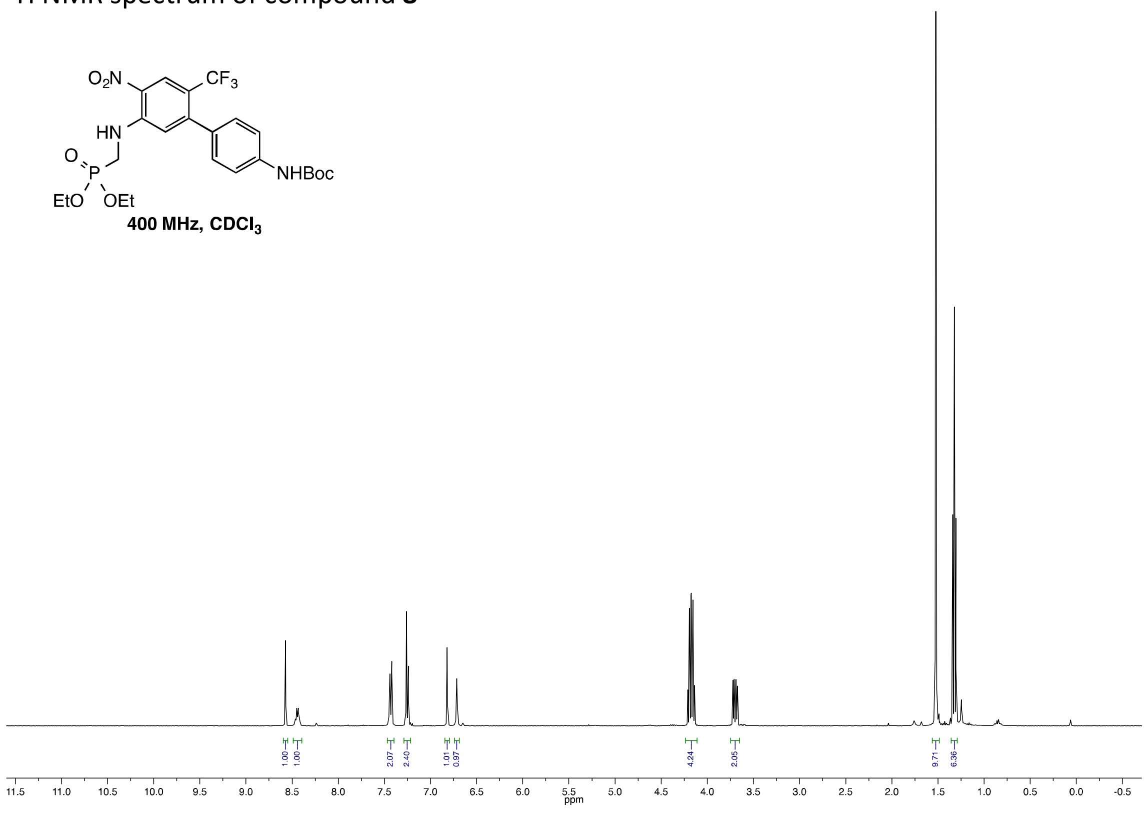

^{13}C NMR spectrum of compound **8**

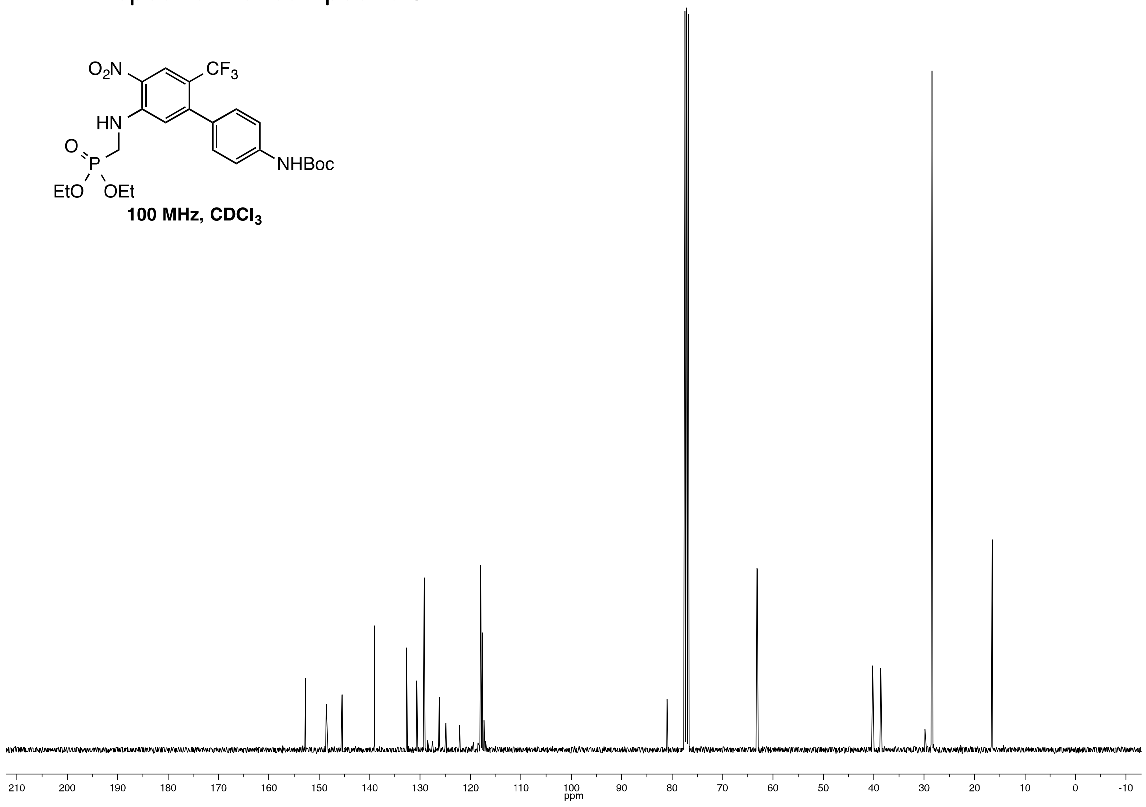

^{1}H NMR spectrum of compound **10**

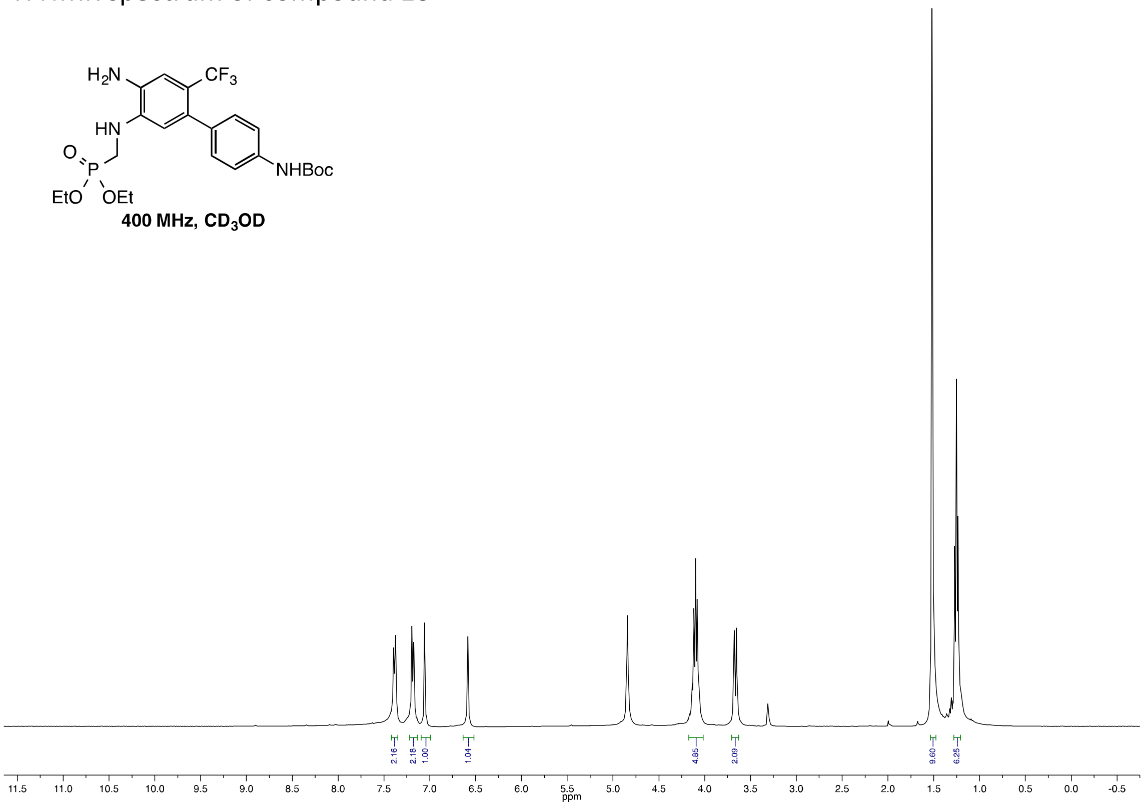

^{13}C NMR spectrum of compound **10**

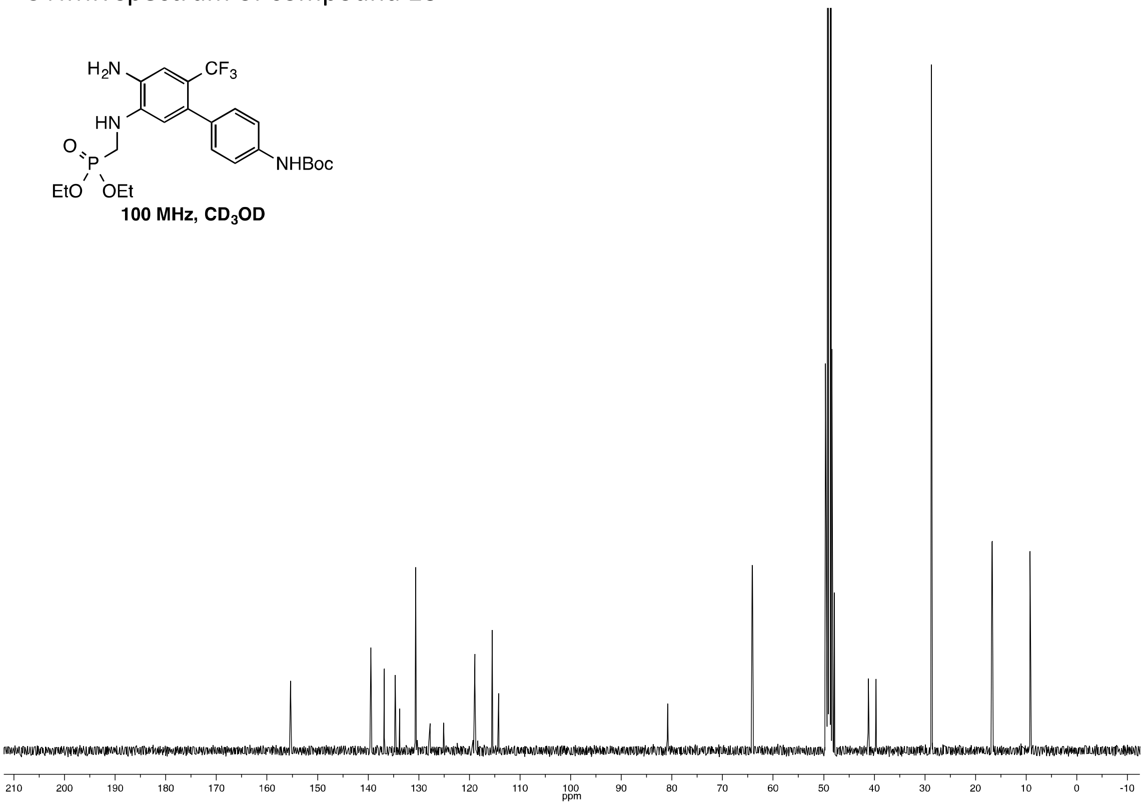

^{1}H NMR spectrum of compound **13**

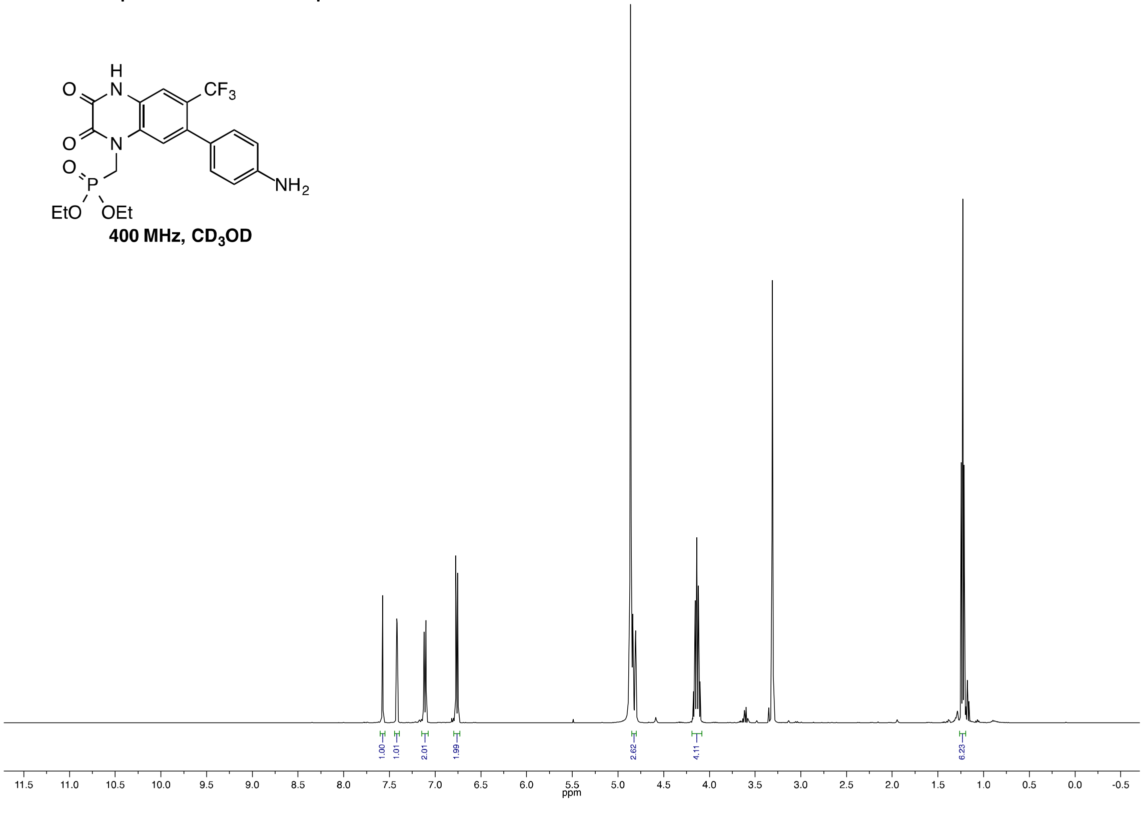

^{13}C NMR spectrum of compound **13**

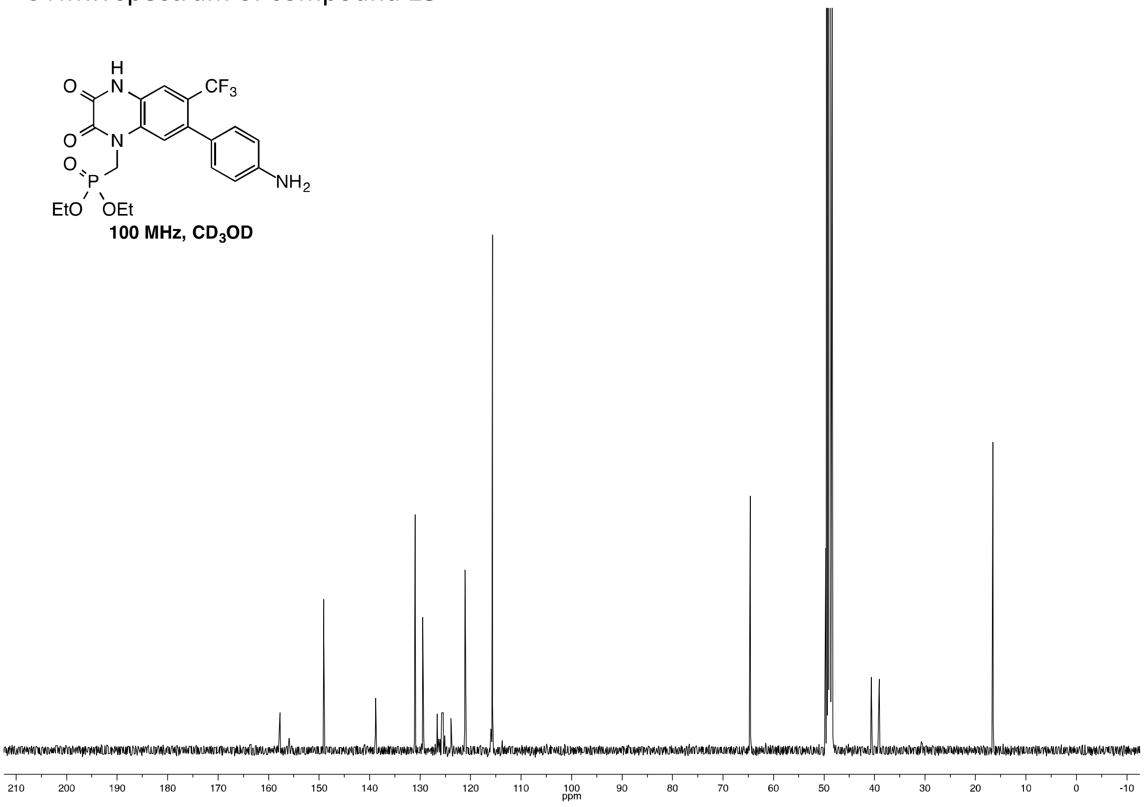

^{1}H NMR spectrum of compound **16**

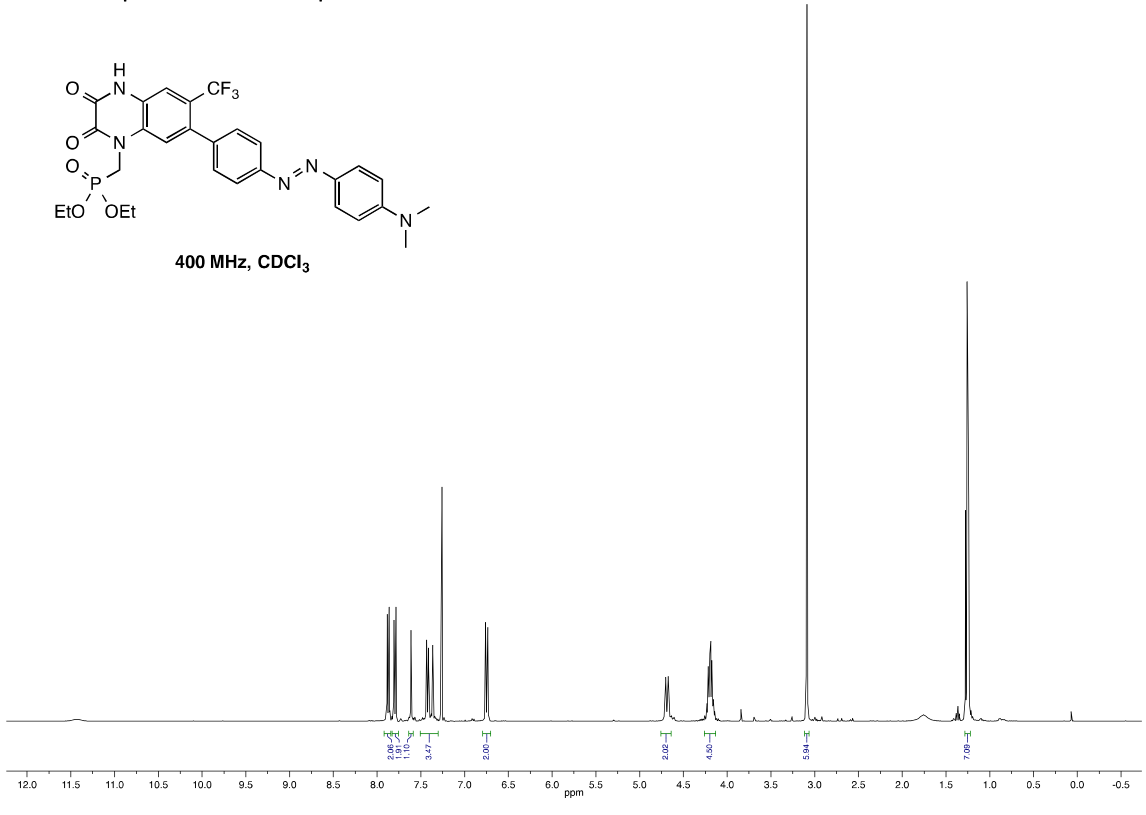

^{13}C NMR spectrum of compound **16**

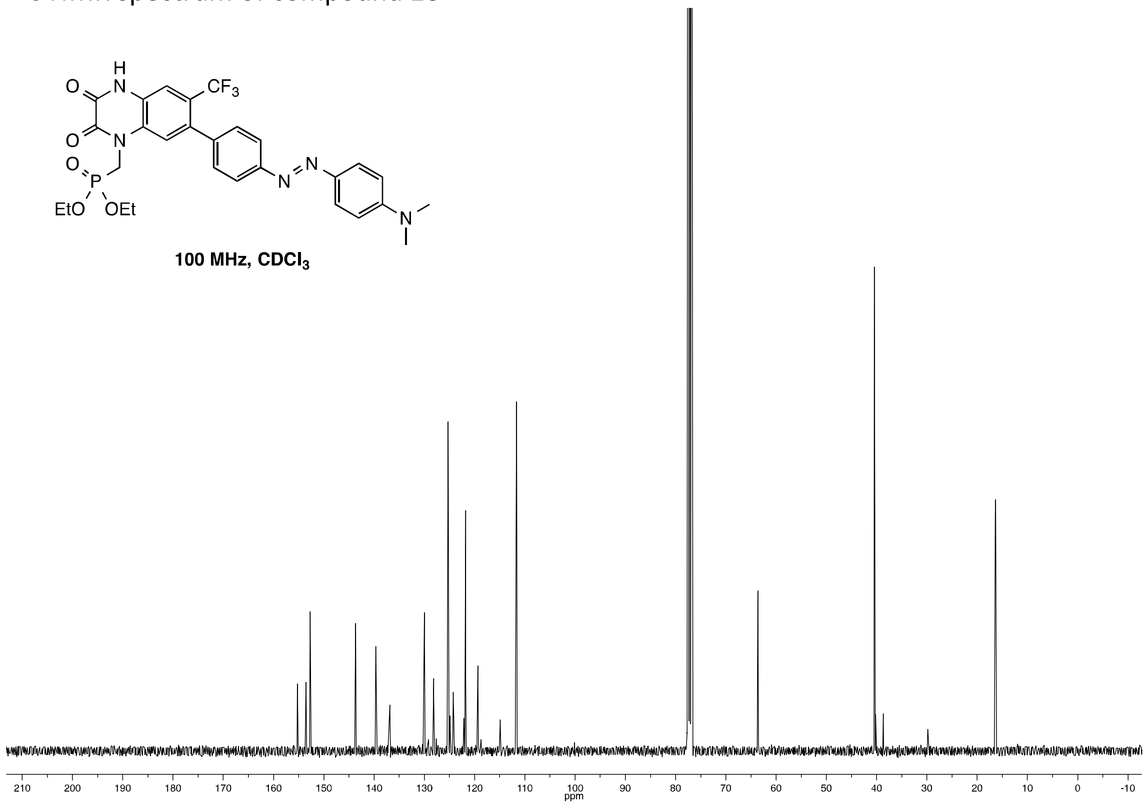

^{1}H NMR spectrum of compound **ShuBQX-2**

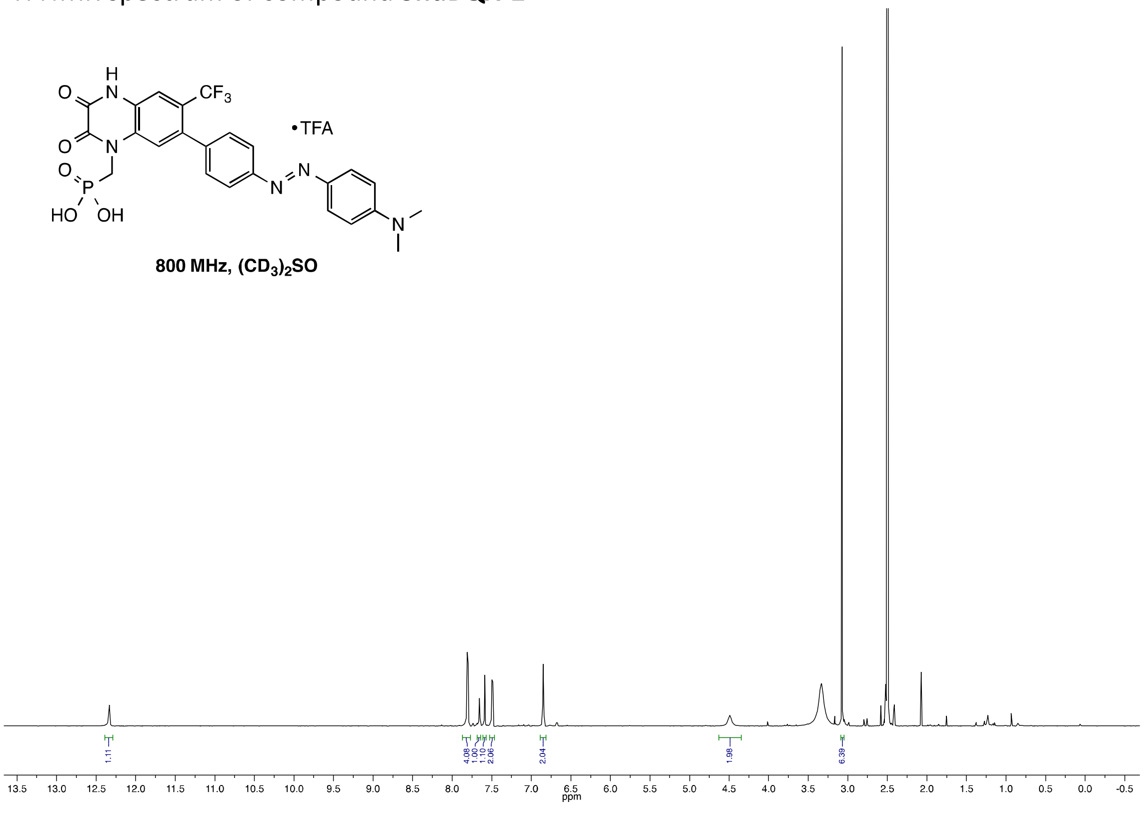

^{13}C NMR spectrum of compound **ShuBQX-2**

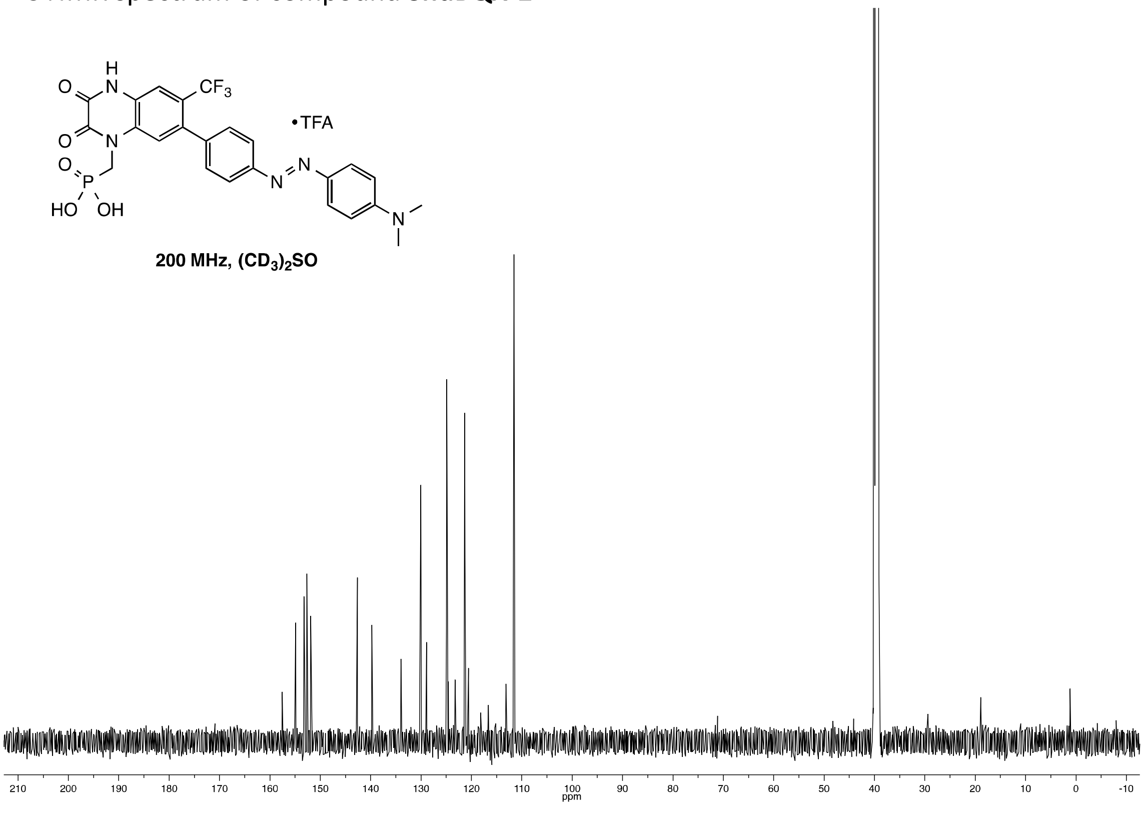

^{1}H NMR spectrum of compound **18**

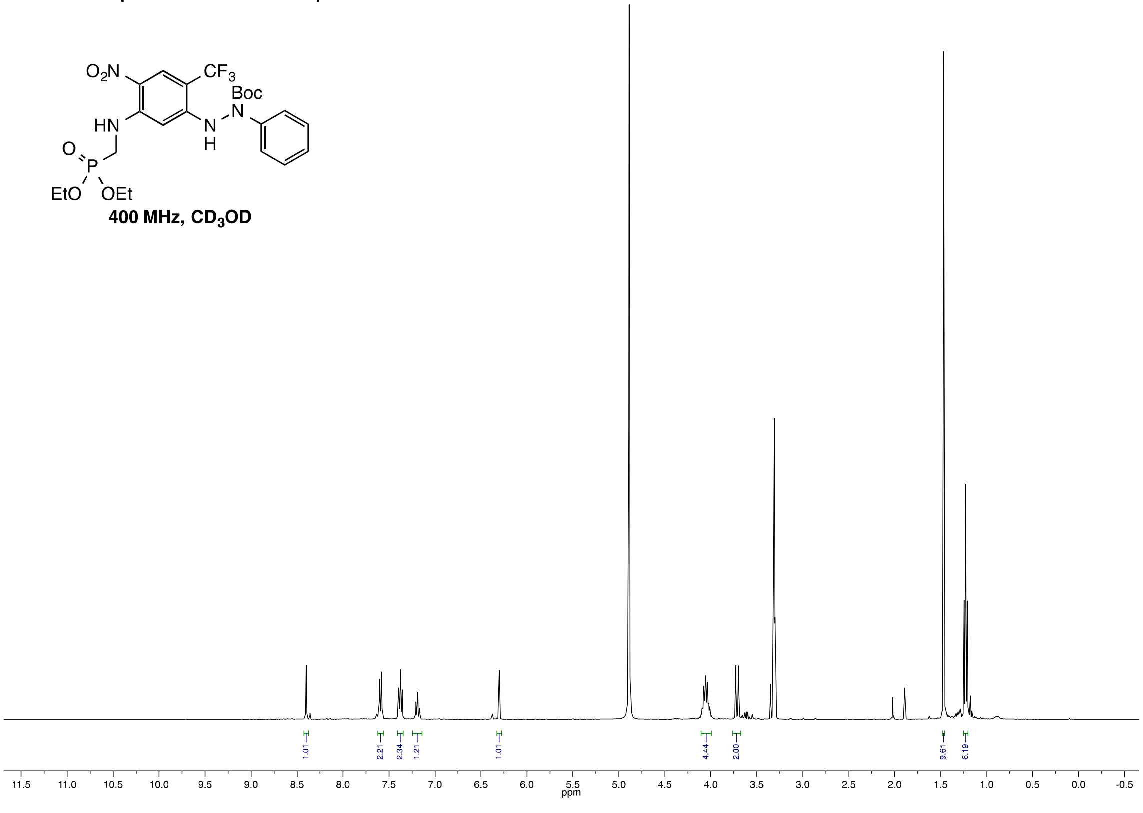

^{13}C NMR spectrum of compound **18**

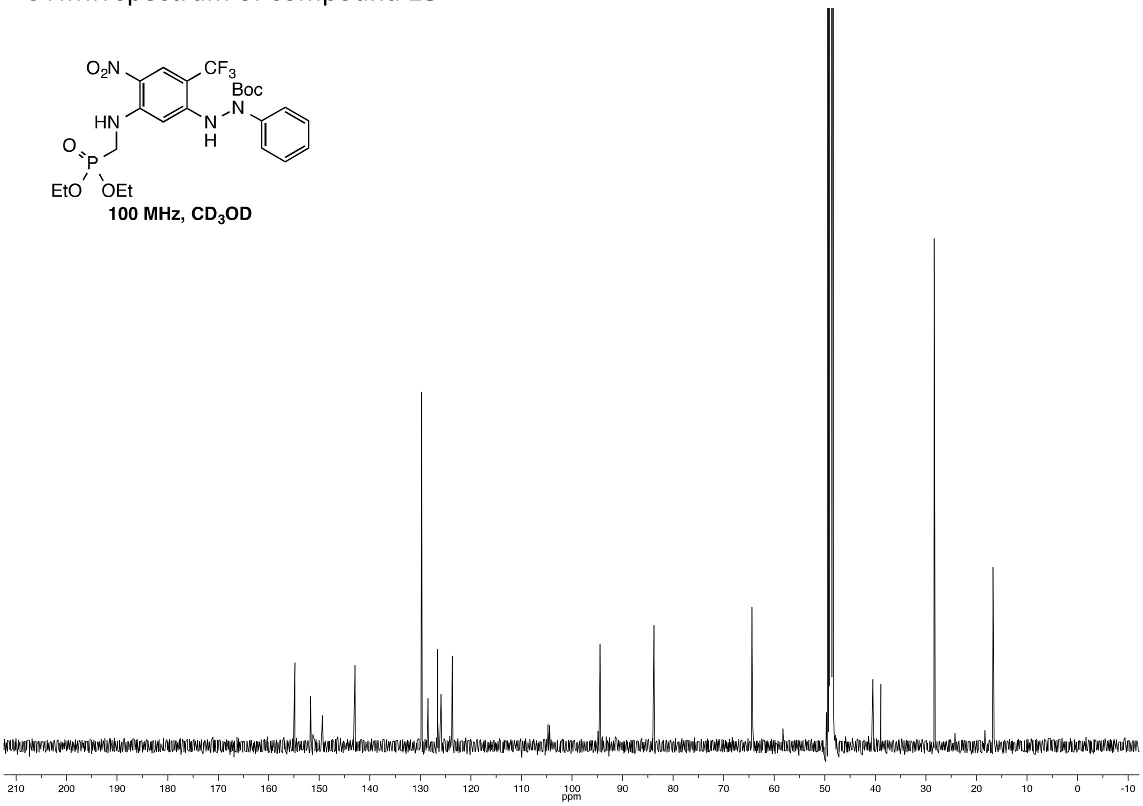

^{1}H NMR spectrum of compound **19**

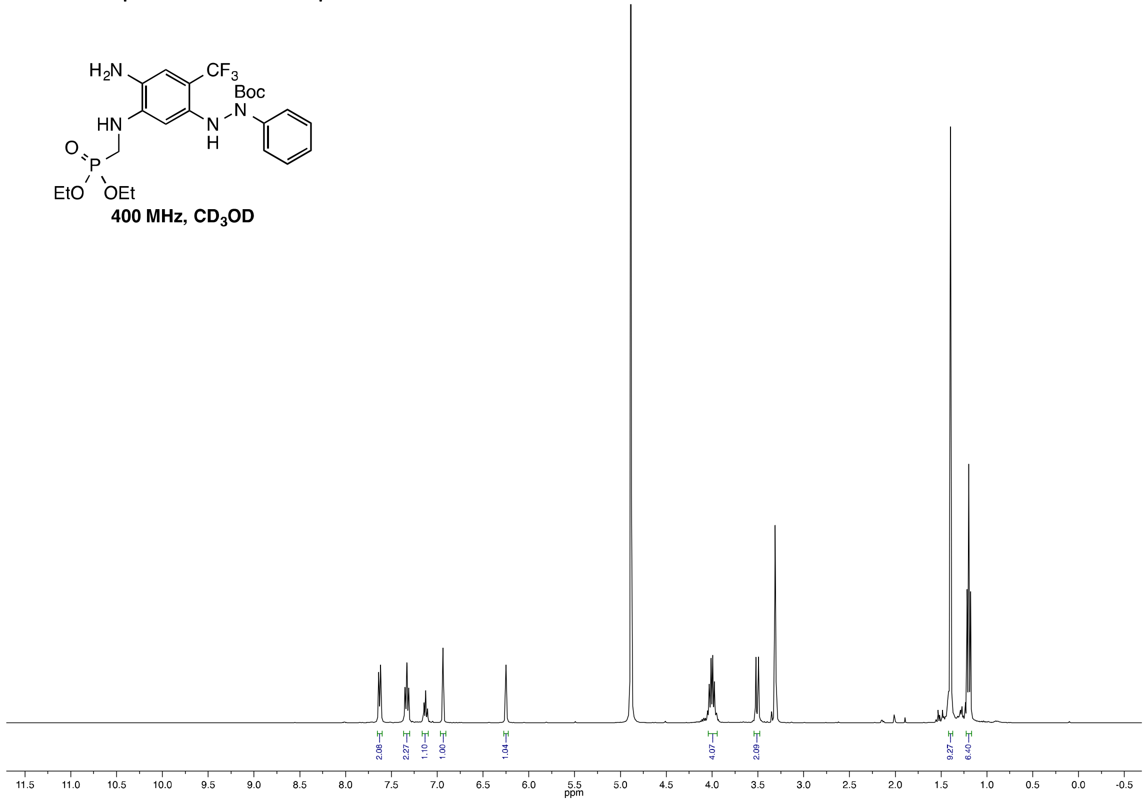

^{13}C NMR spectrum of compound **19**

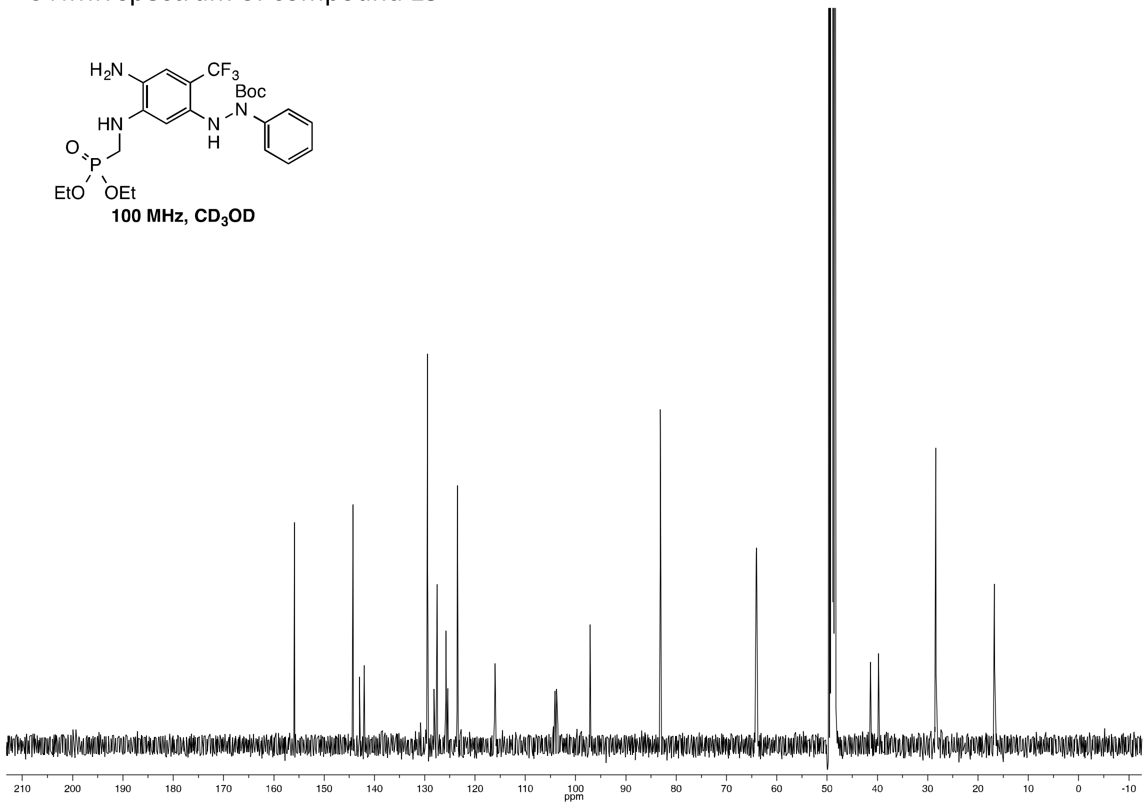

^{1}H NMR spectrum of **S1**

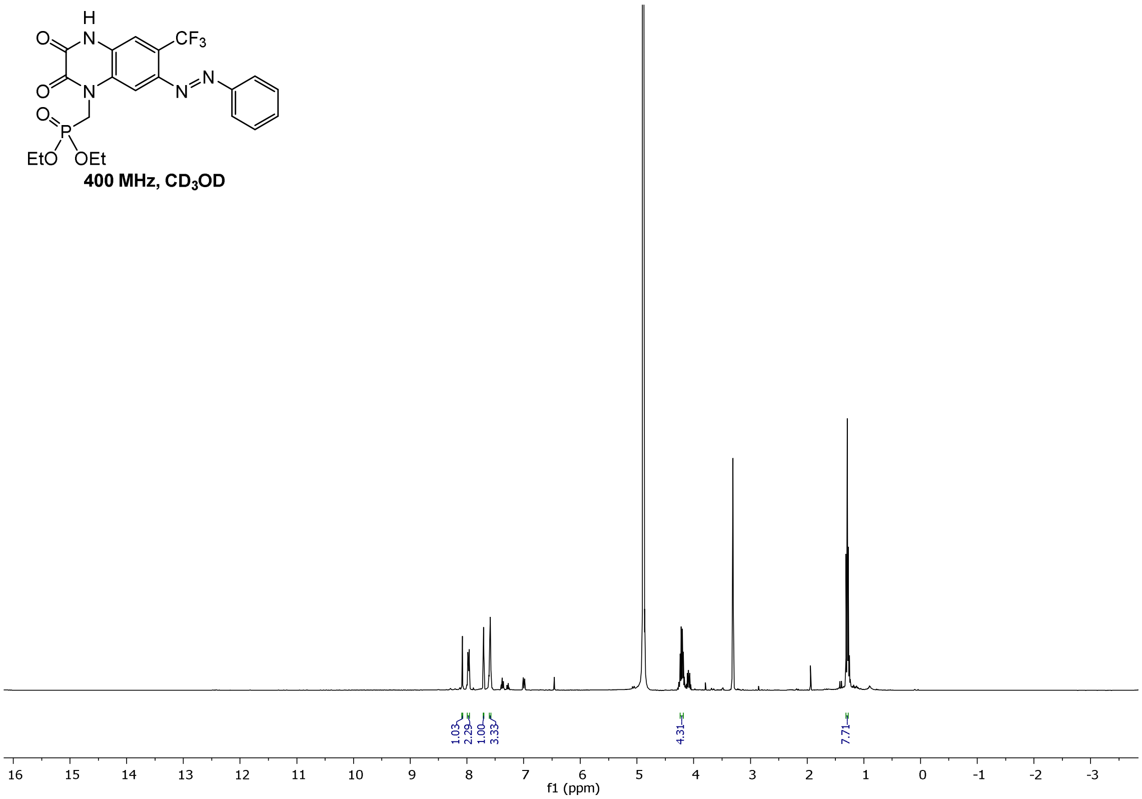

^{13}C NMR spectrum of **S1**

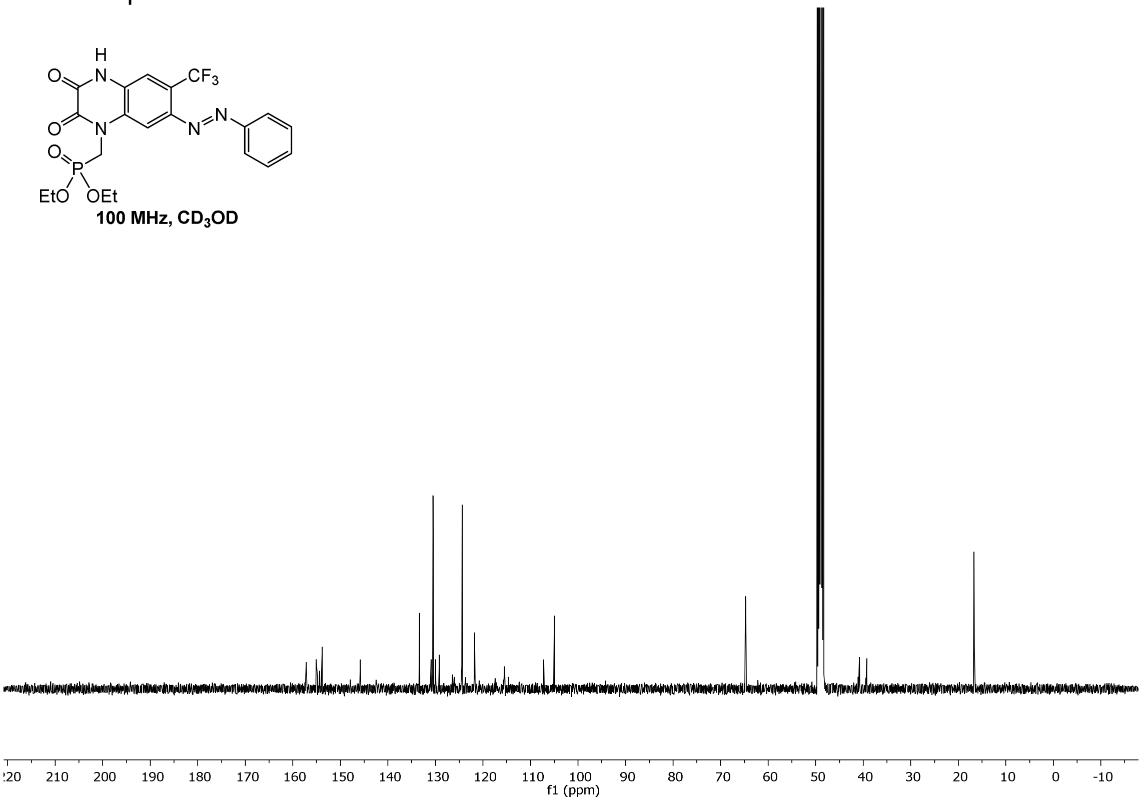

^{1}H NMR spectrum of **ShuBQX-3**

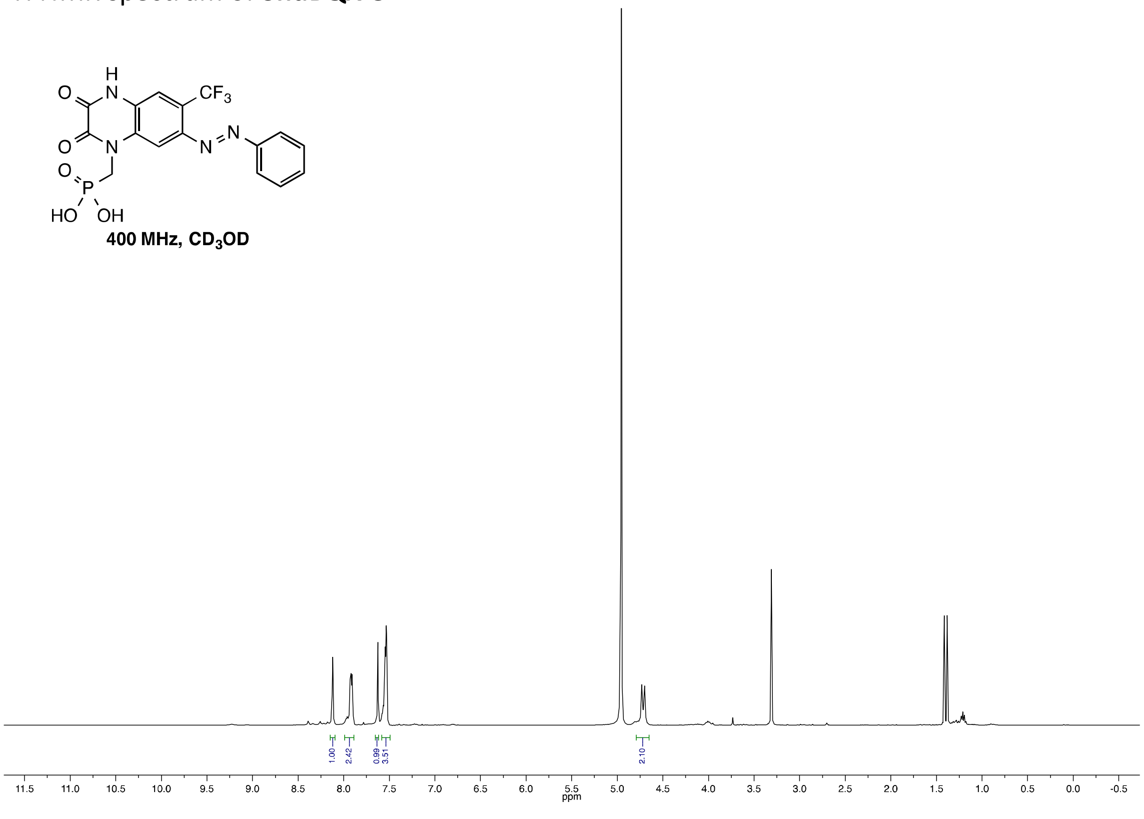

^{13}C NMR spectrum of **ShuBQX-3**

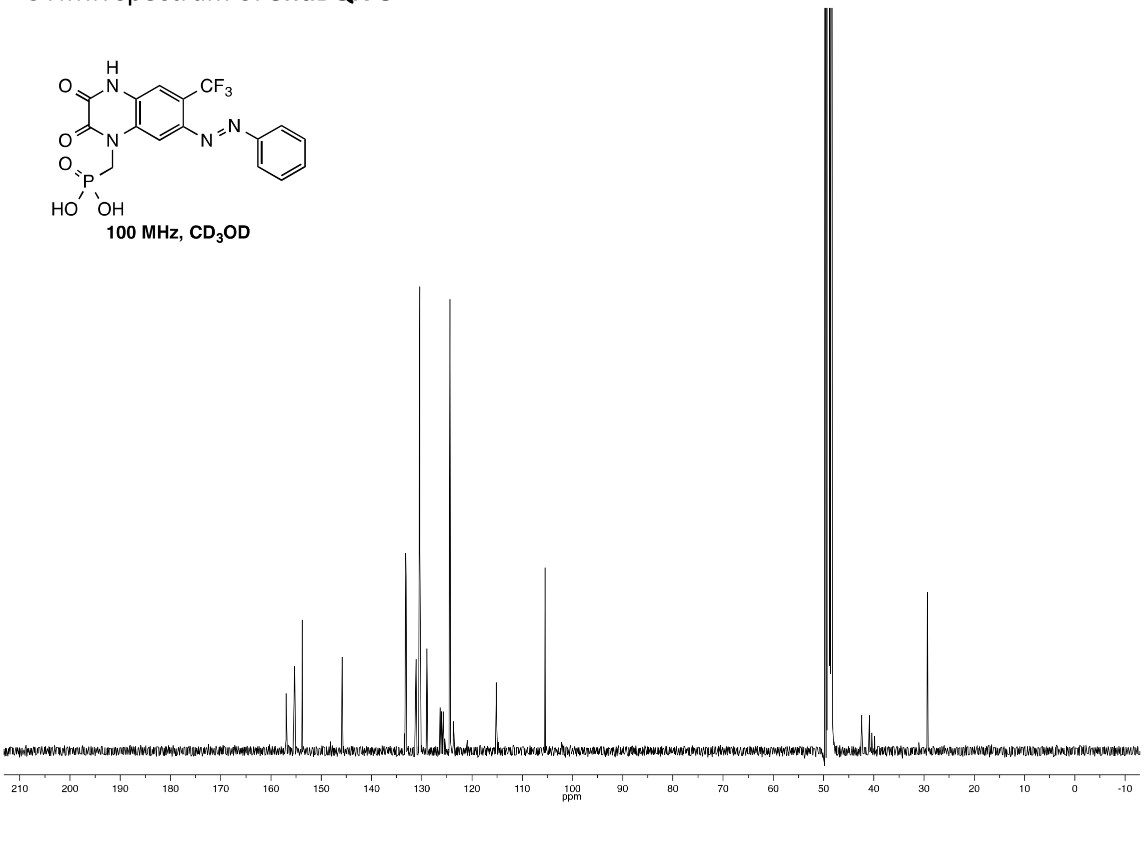

Supplementary Figures

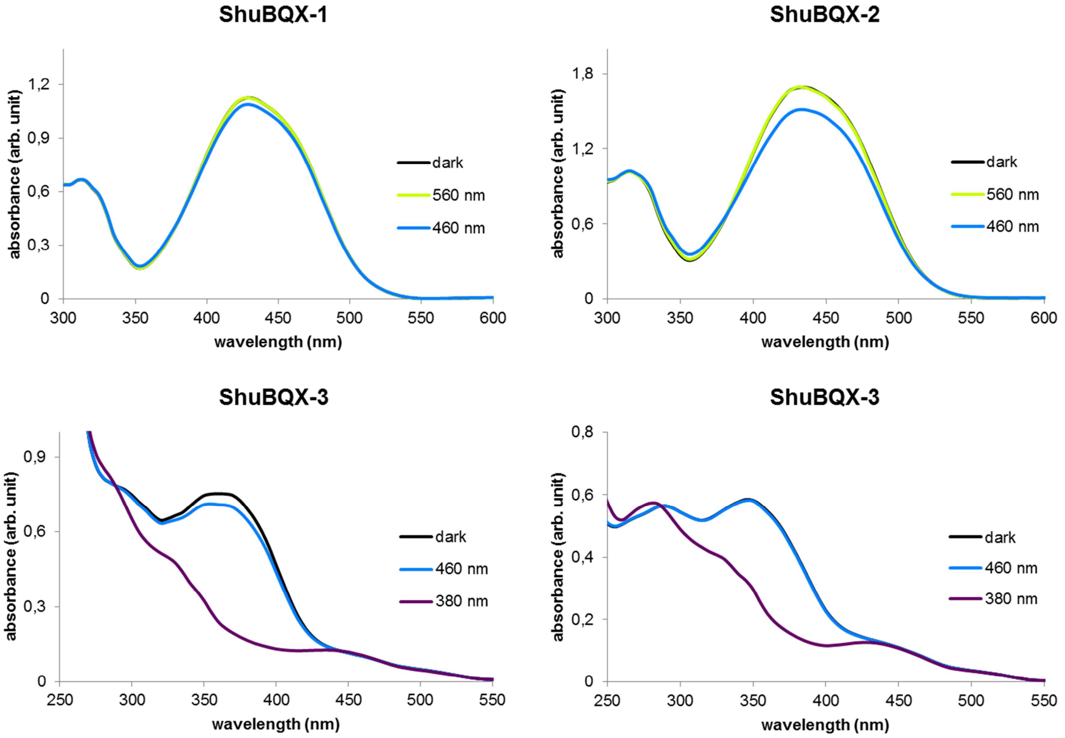

Figure S1. UV-Vis absorption spectra of ShuBQX-1, ShuBQX-2 and ShuBQX-3. Solutions of **ShuBQX-1** (50 µM in DMSO), **ShuBQX-2** (50 µM in DMSO), **ShuBQX-3** (50 µM in DMSO, left and 50 µM in Ringer buffer, right) were placed in a 1 mL quartz cuvette (10 mm diameter). A light-fibre cable connected to a Till Photonics Polychrome 5000 monochromator was placed in the cuvette until it penetrated the surface of the solution. Illumination was screened from wavelengths 380-600 nm in 20 nm steps (**ShuBQX-1** and **ShuBQX-2**) and 340-500 nm in 20 nm steps (**ShuBQX-3**) going from higher to lower wavelengths. Every wavelength was applied for 5 min before a UV-Vis spectrum was recorded. Illumination conditions that afforded the highest *trans*-isomer and *cis*-isomer enrichment are shown in Figure S1.

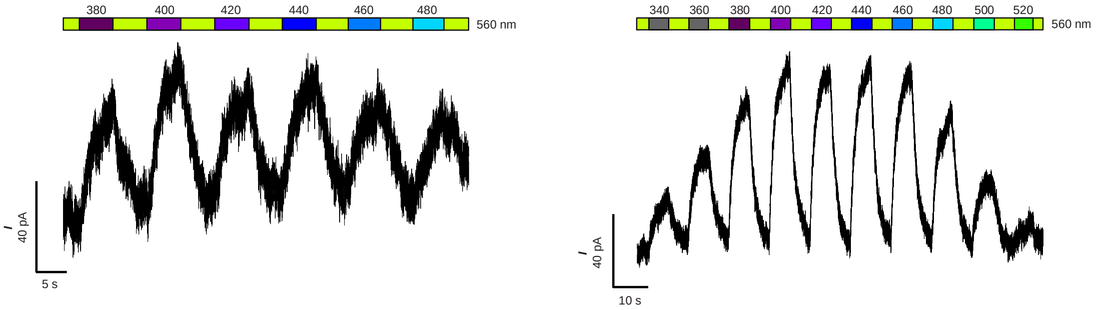

Figure S2. Action spectra of ShuBQX-1 and ShuBQX-2. Action spectra of **ShuBQX-1** (5 µM, left) and **ShuBQX-2** (5 µM, right) in the presence of glutamate (300 µM) under illumination with green light (580 nm) and varying wavelengths.

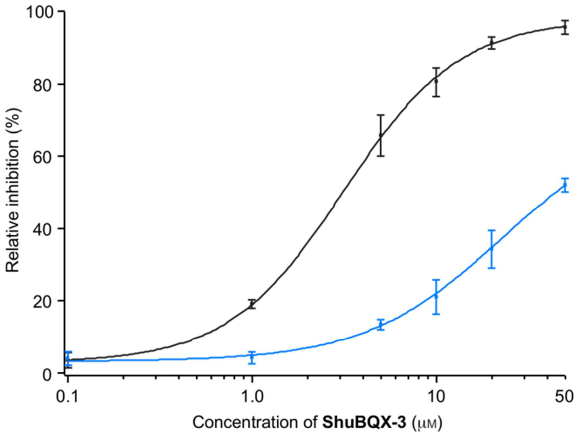

Figure S3. ShuBQX-3 dose-response studies. Normalized dose-response curve of **ShuBQX-3** in the presence of glutamate (300 µM). The *trans*-isomer of **ShuBQX-3** (black, IC_{50} = 3.1 µM) displayed almost full antagonism (95.8% ± 1.8%) of HEK293T cells expressing GluA1-L497Y receptors at 50 µM. The *cis*-isomer of **ShuBQX-3** (blue) was significantly less potent, exhibiting nearly half the antagonism (52.0% ± 1.9%) of the *trans*-isomer. The dose-response curve of **ShuBQX-3** (IC_{50} = 3.3 µM) illuminated with orange light (600 nm) is not shown for clarity. Data points were fitted using the Hill equation from n=5 independent cells. Values represent mean ± SEM.

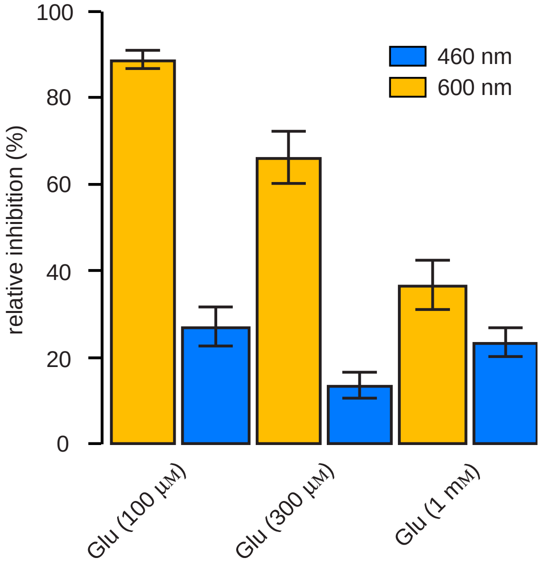

Figure S4. ShuBQX-3 is a competitive antagonist of AMPA receptors. ShuBQX-3 (5 µM) exhibits differing amounts of AMPA receptor antagonism when varying the concentration of glutamate (n=5 cells). Large light-dependent currents were observed when using 100 µM and 300 µM glutamate. Increasing the concentration of glutamate to 1 mM resulted in a significant reduction of light-dependent currents. Values represent mean ± SEM.

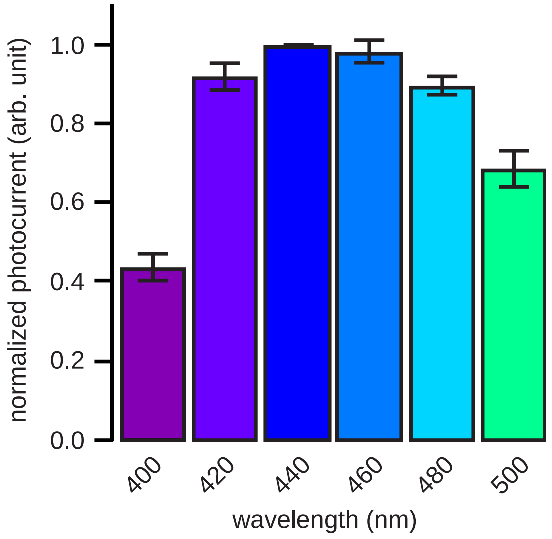

Figure S5. Action spectrum of ShuBQX-3. The current magnitude can be controlled by varying the wavelength of light (400-500 nm) used for photoswitching (**ShuBQX-3**, 20 μM). It was consistently shown that illuminating with blue light (440 nm and 460 nm) provided the maximum inward current (n=5 cells). Values represent mean ± SEM.

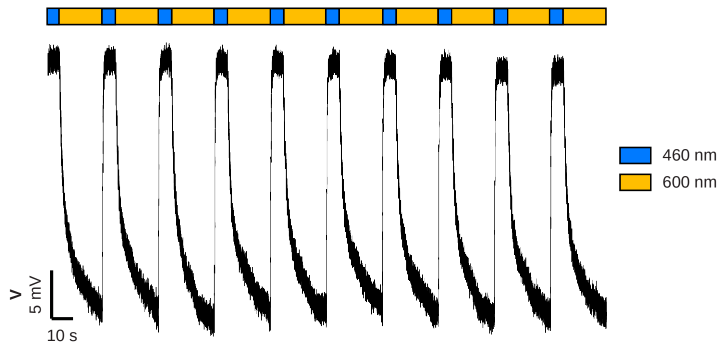

Figure S6. Current-clamp photoswitching of ShuBQX-3. The photoswitching of **ShuBQX-3** (10 μM) was also highly reproducible when in current-clamp mode.

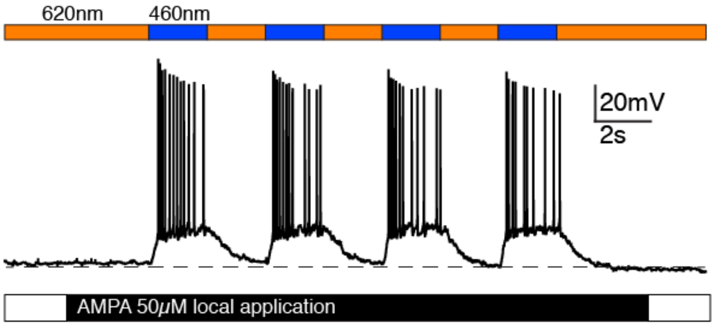

Figure S7. Optical control of action potential firing in hippocampal CA1 neurons using **ShuBQX-3** (10 μM) in the presence of AMPA (50 μM).

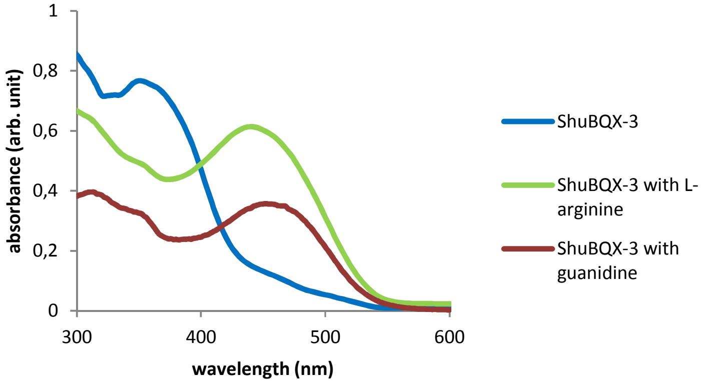

Figure S8. UV-Vis absorption spectrum of ShuBQX-3. Comparison of **ShuBQX-3** (50 μM) alone (blue) and in the presence of 1mM L-arginine (green) and 1 mM guanidine (red) in DMSO. Illumination conditions that afforded the highest *trans*-isomer enrichment are shown.

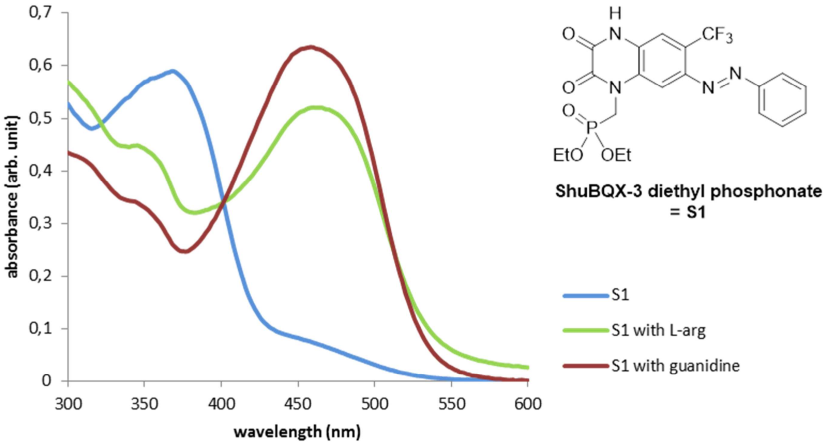

Figure S9. UV-Vis absorption spectrum of ShuBQX-3 diethyl phosphonate (S1). Comparison of **S1** (50 μM) alone (blue) and in the presence of 1mM L-arginine (green) and 1 mM guanidine (red) in DMSO. Illumination conditions that afforded the highest *trans*-isomer enrichment are shown.

HEK293T Cell Electrophysiology

HEK293T cells were incubated in dulbecco´s minimal essential medium + 10% FBS and split at 80 to 90% confluency. For detachment, growth medium was removed, cells were washed with calcium free PBS buffer and cells were treated with trypsin solution at 37 °C for 2 min. Detached cells were diluted with growth medium and singularised by pipetting. For transfection, acid-etched coverslips were coated with poly-L-lysin and placed in a 24-well plate. 40 000 cells were added to each well in 500 µL standard growth medium. DNA (per coverslip: 350 ng GluA1-L497Y and 50 ng YFP) was mixed with 1 µL polyplus jetprime in 50 µL jetprime buffer. After standing at room temperature for 10-15 min, the DNA-mix was added to the cells shortly after seeding them into the abovementioned 24-well plate. After 3-5 hours the medium was exchanged for standard growth medium. Cells were used for electrophysiological recordings 24 hours post transfection.

Whole-cell patch clamp experiments were performed using a standard electrophysiology setup equipped with a HEKA Patch Clamp EPC10 USB amplifier and PatchMaster software (HEKA Electronik). Micropipettes were generated from a Science Products GB200-F-8P with filament pipettes using a vertical puller. Resistance varied between 3-7 MΩ. The extracellular solution contained in mM: 138 NaCl, 1.5 KCl, 2.5 $CaCl_2$, 1.2 $MgCl_2$, 10 glucose and 5 HEPES (NaOH to pH 7.4). The intracellular solution contained in mM: 140 K-gluconate, 5 NaCl, 15 KCl, 5.0 MgATP, 0.5 Na_2ATP and 12.5 HEPES (pH 7.3). The holding potential for voltage clamp experiments was −60 mV. The antagonists **ShuBQX-1**, **ShuBQX-2** and **ShuBQX-3** were diluted into the extracellular solution from 100 mM DMSO stock solutions. Glutamate was diluted into the extracellular solution from a 100 mM H_2O stock solution. Illumination during electrophysiology experiments was provided by a Poly V, FEI monochromator.

Brain Slice Electrophysiology

All animal procedures were performed in accordance with the guidelines of the Regierung Oberbayern. Horizontal slices were prepared from C57Bl6JRj mice (postnatal day 12-14). Following decapitation, the brain was rapidly removed and transferred to an ice-cold saline solution composed of (in mM) 87 NaCl, 75 sucrose, 25 $NaHCO_3$, 2.5 KCl, 1.25 NaH_2PO_4, 0.5 $CaCl_2$, 7 $MgCl_2$, 25 glucose saturated with carbogen (95% O_2/5% CO_2). Slices (300 µm thick)

were cut with a vibratome (NPI Electronic), incubated at 34 °C for 1 h in saline solution and then kept at room temperature for up to 6 h before being used in patch-clamp recordings. Experiments were carried out in ACSF composed of (in mM) 125 NaCl, 26 $NaHCO_3$, 2.5 KCl, 1.25 NaH_2PO_4, 2 $CaCl_2$, 1 $MgCl_2$, 20 glucose and AP-5 (50 µm) saturated with carbogen at room temperature.

Pyramidal CA1 neurons of the hippocampus were patched using glass electrodes (Science Products) with a resistance of 6–9 MΩ. Current-clamp recordings were carried out using the following intracellular solution (in mM): 140 K-gluconate, 10 HEPES, 12 KCl, 4 NaCl, 4 MgATP, 0.4 Na_2GTP. Recordings were made with an EPC 10 USB amplifier, controlled by the Patchmaster software (HEKA). Data was filtered at 2.9 and 10 kHz. Data was analyzed using the Patcher's Power Tools (MPI Göttingen) and IgorPro (Wavemetrics). **ShuBQX-3** (10 µM) together with either (*RS*)-AMPA (50 µM) or glutamate (100 µM) dissolved in ACSF were locally applied through a glass pipette using a pressure application system at 2 psi (NPI Electronic). Photoswitching was achieved through a microscope coupled monochromator (Poly V, FEI).

Xenopus Oocytes Electrophysiology

cRNA was synthesized from cDNA clones of GluN1-1a (GenBank accession number U08261), GluN2A (NM_012573), NR2B (U11419), NR2C (NM_012575), NR2D (NM_022797), GluA1(Q)flip (P19490.2), GluA1(Q)flop (P19490.1), GluA2(R)flip (P19491.2), and GluK2(P42260) subcloned in the *X. laevis* oocyte expression vector pSGEM using the T7 mMESSAGE mMACHINE Kit (Ambion). Synthesized cRNA was isolated via the Clean & Concentrator 25 kit (Zymo), the quality of the cRNA controlled via denaturating agarose gel electrophoresis and the concentration, after photometrical determination, adjusted to 400 $ng \cdot \mu L^{-1}$ with nuclease-free water.

All animal procedures were performed in accordance with the Tierschutzgesetz (TierSchG) and the TierschutzVersuchstierverordnung (TierSchVersV). Frog oocytes of stages V or VI were obtained by surgical removal of the ovaries of a *X. laevis* frog previously anesthetized with ethyl 3-aminobenzoate methanesulfonate (2.3 $g \cdot L^{-1}$; Sigma). The ovaries were cut and under constant shaking incubated with 300 $U \cdot mL^{-1}$ (10 $mg \cdot mL^{-1}$) collagenase type I

(Worthington Biochemicals) for 3 h at 21 °C in Ca^{2+}-free Barth's solution (in mM: 88 NaCl, 1.1 KCl, 2.4 $NaHCO_3$, 0.8 $MgSO_4$, 15 HEPES, pH adjusted to 7.6 with NaOH). The Ca^{2+}-sensitive collagenase reaction was stopped by rinsing with Barth's solution with (in mM: 88 NaCl, 1.1 KCl, 2.4 $NaHCO_3$, 0.8 $MgSO_4$, 0.4 $CaCl_2$, 0.3 $Ca(NO_3)_2$, 15 HEPES, pH adjusted to 7.6 with NaOH). Oocytes were kept in Barth's medium supplemented with 100 $\mu g \cdot mL^{-1}$ gentamycin, 40 $\mu g \cdot mL^{-1}$ streptomycin, and 63 mg/mL penicillin. Oocytes of stages V or VI were selected and injected with cRNA within 8 h after surgery using a Nanoliter 2010 injector (WPI).

For expression of GluA1(Q)flip and GluA1(Q)flop homomeric and GluA1(Q)flip / GluA2(R)flip heteromeric receptors, 4 ng (25 nL) cRNA per subunit were injected. To express the GluK2(Q) homomeric receptor, 4 ng (20 nL) cRNA was injected. For the expression of the heteromeric NMDA receptors, 4 ng (10 nL) GluN1-1a cRNA were coinjected with one of the following: 7 ng (17 nL) GluN2A, or 6 ng (15 nL) GluN2B, or 5 ng (13 nL) GluN2C, or 5 ng (13 nL) GluN2D.

Electrophysiological recordings were performed on days 4-7. Two-electrode voltage clamping was performed using a TurboTec-10CX amplifier (npi electronic) controlled by Pulse software (HEKA). Borosilicate glass capillaries (Harvard Instruments) were pulled to resistances of 0.1-1.0 MΩ and filled with 3 M KCl. Oocytes were clamped at 70 mV. All recordings were performed in barium ringer (BaR, in mM: 115 NaCl, 2.5 KCl, 1.8 $BaCl_2$, 10 HEPES-NaOH, pH 7.2) supplemented with 250 mM niflumic acid (NFA) to prevent the opening of endogenous calcium-induced chloride channels. For the recording of kainate receptor currents either 100 μM or 30 μM glutamate were used, for the recording of AMPA receptor currents 100 μM kainic acid, and for the recording of the NMDA receptor currents 100 μM glutamate / 10 μM glycine. Also, agonist solutions additionally containing 5 μM **ShuBQX-3** were prepared. For the recording of the **ShuBQX-3**-mediated block of current responses, agonist solution without **ShuBQX-3** was perfused at least for 10 s until a steady state was reached, followed by a 10 s application of agonist solution containing 5 μM **ShuBQX-3**. After a subsequent second application of agonist solution without **ShuBQX-3** a washout with BaR without agonist followed. The **ShuBQX-3**-mediated block was then calculated as the **ShuBQX-3**-induced percent inhibition of the total agonist-induced current response.
